# Physics-guided thermodynamic screening of H_2_ adsorption on alkaline-earth-modified anatase TiO_2_ nanoparticles with LLM-assisted interpretation

**DOI:** 10.1039/d6ra04252j

**Published:** 2026-10-07

**Authors:** Mustafa Kurban, Can Polat, Erchin Serpedin, Hasan Kurban

**Affiliations:** a Department of Electrical and Computer Engineering, Texas A&M University at Qatar Doha Qatar kurbanm@ankara.edu.tr; b Department of Prosthetics and Orthotics, Ankara University Ankara Türkiye; c Department of Electrical and Computer Engineering, Texas A&M University College Station TX USA; d College of Science and Engineering, Hamad Bin Khalifa University Doha Qatar hkurban@hbku.edu.qa

## Abstract

Understanding how surface modification tunes H_2_ adsorption on oxide nanomaterials is important for designing reversible hydrogen-interaction platforms under near-ambient conditions. Here, we examine single-H_2_ adsorption on a fixed, idealized ∼1.8 nm anatase-derived TiO_2_ nanoparticle reference model, both pristine and surface-modified with alkaline-earth (AE) species (Be, Mg, Ca, Sr, Ba, and Ra), using GATOR, a physics-guided DFTB-based screening framework with LLM-assisted interpretation. Within this framework, the deterministic thermodynamic screening sequence defines the classification, whereas the constrained language model serves only as an interpretive layer rather than as a predictor of adsorption physics. The workflow combines DFTB-derived adsorption energetics, adsorption-mode analysis, finite-temperature thermodynamic descriptors, Langmuir-type coverage estimates, rule-based thermodynamic screening, and post-hoc consistency checks to evaluate local H_2_ uptake-release behavior. Electronic adsorption energies are mapped onto finite-temperature descriptors at 298 K, including finite-temperature-corrected adsorption enthalpy surrogates, standard-state free-energy estimates, desorption midpoint temperatures, and pressure-dependent fractional coverages. Across the series, Be–TiO_2_ and Ra–TiO_2_ occupy excessive-binding/heating-needed regimes, whereas Ca-, Sr-, and Ba-modified TiO_2_ remain weak-binding and under-loaded near ambient pressure. Pristine TiO_2_ and Mg-modified TiO_2_ represent the most balanced strong-binding cases, but both lie above the practical desorption window under the entropy-consistent thermodynamic treatment. Frontier-orbital and conceptual-DFT descriptors change only modestly upon H_2_ adsorption, indicating that the observed trends are governed mainly by local adsorption geometry and finite-temperature adsorption thermodynamics rather than by substantial global electronic-structure reorganization. Within the DFTB-based screening framework, pristine TiO_2_ and Mg–TiO_2_ occupy the most balanced region of the representative adsorption landscape. Higher-level PBE-D3(BJ) calculations support attractive local H_2_ interactions for both representative motifs, but they do not reproduce the small DFTB energetic preference for Mg–TiO_2_ and place the local Mg–H_2_ minimum at a longer separation. Accordingly, the pristine/Mg ordering should be regarded as method-sensitive, and no method-independent energetic preference between these two systems is assigned.

## Abstract 2

Understanding how surface modification tunes H_2_ adsorption on oxide nanomaterials is important for designing reversible hydrogen-interaction platforms under near-ambient conditions.

## Graphical abstract

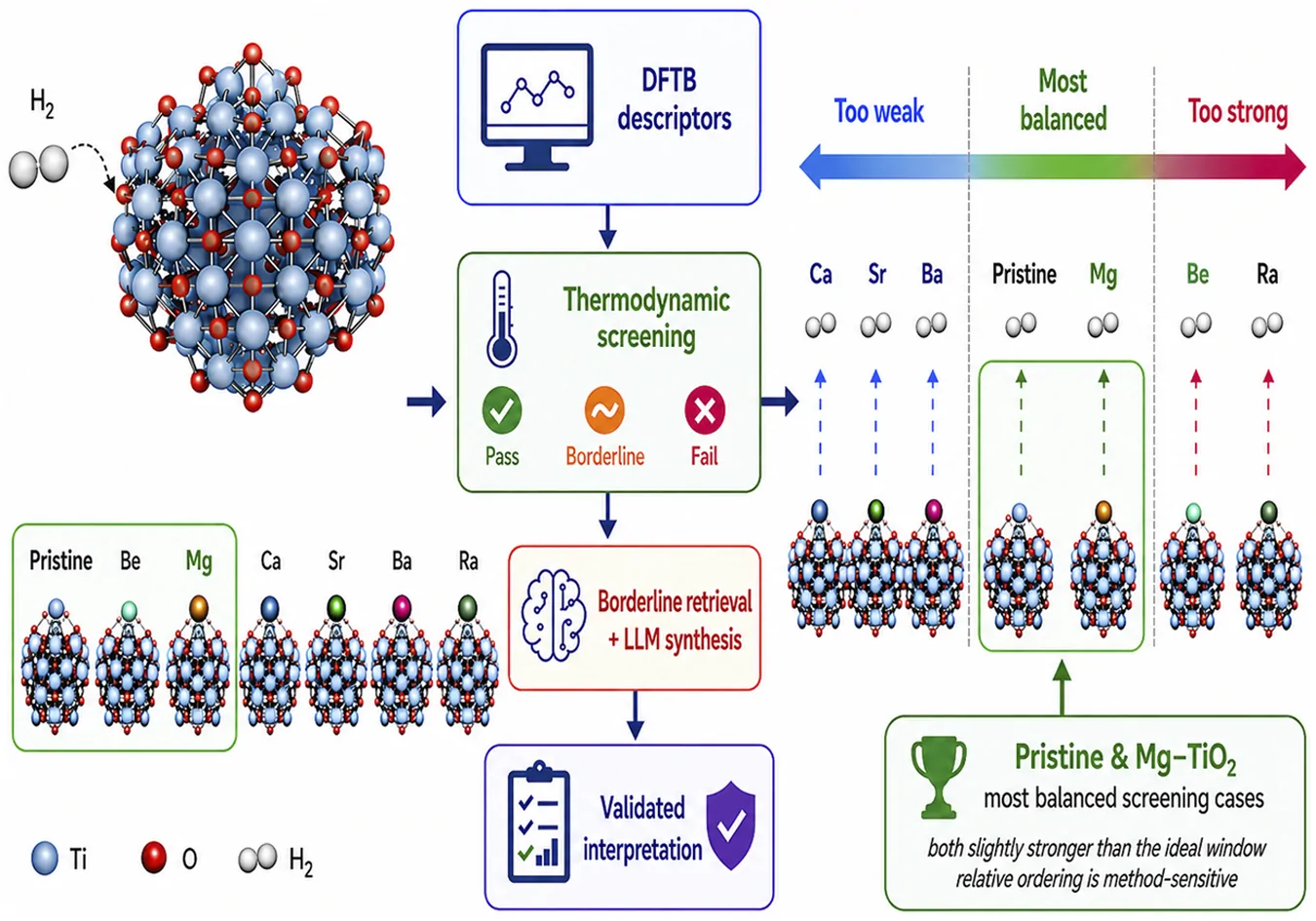

## 1 Introduction

Hydrogen is central to many net-zero energy scenarios, yet practical materials-based storage remains constrained by a simple thermodynamic requirement: the interaction with H_2_ must be strong enough to enable uptake near ambient conditions, but weak enough to permit release without large energetic penalties. Achieving this balance is particularly challenging in solid-state adsorbents, where weak physisorption suppresses room-temperature loading, whereas stronger binding can compromise reversibility and deliverability. For this reason, an adsorption enthalpy of roughly 15–25 kJ mol^−1^ per H_2_ is widely regarded as a practical target for near-ambient reversible storage.^1,2^ This target is also closely linked to the substantial entropy loss of gas-phase H_2_ upon adsorption, which makes reversible operation around 298 K intrinsically demanding.^3,4^

Among oxide materials, TiO_2_ is an attractive platform for hydrogen-related surface chemistry because it is abundant, chemically robust, and capable of supporting adsorption, activation, and spillover-mediated hydrogen processes. Its surface reactivity is highly sensitive to crystal phase, surface termination, and local defect structure, and anatase often provides a more favorable platform than rutile for moderate hydrogen interaction.^5–7^ More broadly, the strong dependence of hydrogen behavior on local coordination suggests that targeted surface modification may offer a practical route for tuning H_2_ adsorption energetics on oxide nanostructures.^8,9^

Alkaline-earth (AE) surface modification is especially interesting in this context. AE species can perturb the local Ti–O electronic environment, alter surface acidity/basicity, and reshape adsorption sites relevant to hydrogen interaction. Mg-, Ca-, Sr-, and Ba-modified TiO_2_ systems have already been explored in photocatalytic and adsorption-related settings, with evidence that Mg in particular can moderate defect-related electronic structure and promote more balanced surface reactivity.^10,11^ More broadly, recent descriptor-guided screening of H_2_ adsorption on single-atom-doped anatase TiO_2_ nanoparticles has shown that local surface modification can tune adsorption energetics and uptake-release behavior across chemically distinct adsorption environments.^12^ However, systematic comparisons of H_2_ adsorption across the AE series on finite anatase TiO_2_ nanoparticles remain limited, especially when the problem is framed not in terms of photocatalytic activity or total storage capacity, but in terms of local uptake-release thermodynamics under near-ambient conditions.^10,13^

That distinction is important. High-surface-area adsorbents such as MOFs are typically assessed through gravimetric or volumetric capacity, whereas the present problem is more local and more fundamental: can the binding of a single H_2_ molecule on a chemically robust oxide nanoparticle be tuned into the near-ambient reversible window? In this study, we address that question using a fixed ∼1.8 nm anatase TiO_2_ nanoparticle model, considered both in pristine form and after AE surface modification. The selected particle should be viewed as an idealized anatase-derived crystalline reference rather than as a claim for the equilibrium structure or morphology of an isolated 1.8 nm TiO_2_ particle. Titania is known to exhibit pronounced size-dependent structural evolution. Lamiel-Garcia *et al.* predicted the emergence of anatase-like crystallinity at approximately 2–3 nm and showed that, at smaller sizes, bulk-cut anatase nanocrystals can be metastable relative to non-crystalline nanoclusters.^14^ Subsequent work from the same group explicitly demonstrated strong size dependence in realistic anatase nanoparticle models spanning the 1–6 nm range.^15^ Experimental observations are consistent with this structural crossover: crystalline anatase particles have been reported at approximately 4–5 nm,^16,17^ whereas TiO_2_ particles in the approximately 2–3 nm regime can exhibit X-ray-amorphous structures containing only a strained anatase-like core and a strongly distorted outer shell.^18^ Accordingly, the present 1.8 nm model is deliberately employed as a controlled crystalline scaffold for isolating the chemical effect of AE modification at fixed particle size and topology; it is not intended to reproduce the complete size- or morphology-dependent phase behavior of experimental TiO_2_ nanoparticles. By keeping the nanoparticle topology constant, we isolate the effect of chemical modification on adsorption strength, adsorption mode, and finite-temperature operability. We therefore evaluate not only the electronic adsorption energy *E*_ads_, but also finite-temperature screening descriptors including corrected adsorption enthalpy surrogates, standard-state free energies, desorption midpoint temperatures, and Langmuir-model coverages, thereby linking atomistic energetics to experimentally relevant uptake-release behavior.^3,19^

The objective of this work is not to predict full storage capacity for a realistic particulate material, but rather to establish a surface-science-based screening picture of how AE modification shifts local H_2_ binding on anatase TiO_2_. Within this framework, we provide a comparative map of adsorption strength, reversibility, and deliverability across the AE series, and identify which modifications move oxide-based H_2_ adsorption closest to the desired near-ambient operating regime. Methodologically, the screening is organized as an integrated, reproducible pipeline in which deterministic physics gates make every pass, borderline, or fail decision, while a constrained large language model serves only as an interpretive layer—providing fact-checked comparison, ranking, and triage under fixed gate constraints rather than predicting adsorption physics. The methodological contribution is this integrated pipeline as a whole, not any individual model output.

## 2 Methodology

### 2.1 Density-functional tight-binding (DFTB) calculations

#### 2.1.1 Structural models

The anatase TiO_2_ nanoparticle model was constructed by replicating the anatase unit cell into a 30 × 30 × 30 supercell, from which a finite spherical nanoparticle with an approximate radius of 0.9 nm (corresponding to a diameter of ∼1.8 nm) was carved. The spherical construction is used here as a controlled top-down reference geometry and not as an experimentally assigned equilibrium morphology at this particle size. In particular, the approximately 1.8 nm diameter lies at or below the reported size regime in which fully developed anatase crystallinity becomes thermodynamically preferred; theoretical estimates place the non-crystalline–crystalline crossover near 2–3 nm, while experimentally characterized particles in this ultrasmall regime may retain only short-range anatase-like order within a substantially distorted or amorphous structure.^14,18^ The nearly spherical initial cut was selected so that the pristine and AE-modified systems could be compared on exactly the same finite parent topology without introducing a simultaneous change in particle size or facet population. Importantly, no constraint was imposed to preserve spherical symmetry during structural optimization; “spherical” refers only to the initial carving procedure, and all nanoparticles were subsequently relaxed without symmetry constraints. Consequently, the present calculations are designed to resolve relative chemical-modification effects within one controlled nanoparticle scaffold, rather than to establish size-dependent adsorption energies or the thermodynamic equilibrium morphology of TiO_2_. The resulting TiO_2_ nanoparticle was treated as a finite nanoparticle in vacuum rather than as a periodically repeated slab. Accordingly, the exposed adsorption environments are more appropriately described as mixed local coordination motifs than as uniquely defined low-index facets. AE-modified systems were generated by placing a single alkaline-earth atom (AE ∈ Be, Mg, Ca, Sr, Ba, Ra) at a surface O-top site, followed by full structural relaxation without symmetry constraints. Importantly, “AE-modified” in the present work denotes a surface-bound AE adatom and not substitutional incorporation of the AE atom into the TiO_2_ lattice. No Ti or O atom was removed or replaced. Thus, all modified systems retain the same 269-atom Ti/O parent framework (89 Ti and 180 O atoms), while the AE species is initially placed above the same representative surface O site. The common initial placement was used only to provide a controlled structural reference across the AE series; neither the AE position nor the TiO_2_ framework was constrained during optimization. Pristine and AE-modified nanoparticles were treated using an identical protocol. The adsorption models were designed to compare chemically analogous local adsorption motifs under a fixed screening protocol rather than to enumerate the complete configurational landscape of every nanoparticle. For the H_2_ adsorption calculations, one representative adsorption configuration was considered for each pristine or AE-modified TiO_2_ nanoparticle. In the AE-modified systems, H_2_ was initially placed near the AE-centered adsorption region, whereas for pristine TiO_2_ it was placed near a representative surface Ti site; the resulting adsorbate-nanoparticle structures were then fully relaxed within the same DFTB workflow. No exhaustive site-and-orientation search over all possible nanoparticle surface sites was performed. Consequently, the ranking reported here should be interpreted as a representative-site ranking obtained under a fixed and protocol-consistent adsorption setup, not as a configurationally converged global ranking over all possible surface sites and H_2_ orientations. In particular, pristine TiO_2_ and Mg–TiO_2_ are identified as the most balanced cases only within this representative single-site screening picture. A multi-site sampling of several alternative pristine and Mg–TiO_2_ adsorption geometries is therefore required before assigning global minimum adsorption energies or site-independent rankings. Therefore, the reported adsorption energies should be interpreted as representative, protocol-consistent screening values for comparing the effect of alkaline-earth modification, rather than as global minimum adsorption energies over the complete configurational landscape. Be and Ra were included only for theoretical contrast and are not considered practical surface modifiers because of the toxicity of Be and the radioactivity of Ra. To quantify whether AE surface modification induces large-scale reconstruction of the TiO_2_ scaffold, the relaxed Ti/O framework of each bare AE-modified nanoparticle was rigidly superposed onto the relaxed pristine nanoparticle using the 269 corresponding Ti and O atoms. After optimal rigid-body alignment, we evaluated (i) the framework root-mean-square displacement (RMSD), (ii) the maximum Ti/O atomic displacement, and (iii) a local RMSD for framework atoms lying within 4.0 Å of the anchoring surface O atom. These quantities distinguish global nanoparticle reconstruction from localized relaxation around the surface-bound AE species.

#### 2.1.2 Hamiltonian and computational parameters

Calculations were carried out with DFTB+.^20^ DFTB was adopted here as a computationally efficient approach for obtaining optimized geometries and adsorption energetics for finite nanoparticle models of this size. All DFTB+ calculations were performed using the PTBP (periodic table baseline parameter) Slater–Koster parameter set.^21^ This choice is important because DFTB is a semi-empirical method and the Hamiltonian and repulsive interactions are defined by the selected Slater–Koster parametrization. PTBP was developed as a broad-coverage solid-state DFTB parametrization for elements up to *Z* ≤ 86 and was fitted against PBE reference data for real and fictitious homoelemental crystals using both energetic and structural targets. Its transferability was further assessed for multicomponent systems, including binary oxides and hydrides, which are directly relevant to the present Ti–O/H/alkaline-earth chemical space. Therefore, PTBP provides a consistent and physically motivated baseline for comparing H_2_ adsorption trends across the pristine and alkaline-earth-modified TiO_2_ nanoparticles within a fixed model chemistry. At the same time, because PTBP is a baseline semi-empirical parametrization rather than a system-specific DFT-level fit, the resulting adsorption energies are interpreted primarily as comparative screening descriptors. To directly assess the method sensitivity of the adsorption physics, the two decision-sensitive representative systems, pristine TiO_2_ and Mg–TiO_2_, were additionally benchmarked using higher-level PBE-D3(BJ) calculations on nested local cluster models, as described below. Long-range dispersion interactions were included using the Grimme D3 correction with Becke–Johnson damping, with three-body terms enabled.^22,23^ Electronic occupations were treated using Fermi smearing at 300 K.

It should also be emphasized that the PTBP set is not an arbitrary or narrowly system-specific Slater–Koster parametrization. In the original PTBP work, the electronic and repulsive parameters were optimized simultaneously using PBE reference data for real and fictitious homoelemental crystals, with energy–volume curves covering different coordination environments as fitting targets. The reported benchmark performance of PTBP was favorable among broad-coverage DFTB models, with average relative equilibrium-volume and energy errors of 2.17% and 75.0 meV atom^−1^, respectively, for the elemental solid-state benchmark. More importantly for the present Ti–O/H/alkaline-earth systems, the transferability of PTBP was explicitly assessed for multicomponent binary materials, including hydrides and oxides. These benchmarks showed that PTBP provides a robust solid-state baseline parametrization rather than a purely molecular or narrowly fitted parameter set. Therefore, although the present DFTB adsorption energies are not claimed to be high-level absolute DFT adsorption enthalpies, they provide a physically motivated and internally consistent basis for comparing adsorption trends across the pristine and alkaline-earth-modified TiO_2_ nanoparticles.

The DFTB-derived adsorption energies are subsequently mapped onto finite-temperature thermodynamic metrics for assessing uptake-release behavior across the alkaline-earth series.

#### 2.1.3 Geometry optimization

All structures were optimized using the rational line-search optimizer until the maximum force component fell below 0.02 eV AÅ^−1^, with a maximum of 3000 optimization steps. No positional constraints were applied. The fully relaxed structures were used in all subsequent analyses.

#### 2.1.4 Higher-level DFT validation

Because pristine TiO_2_ and Mg–TiO_2_ lie closest to the thermodynamic screening boundaries and are therefore the systems for which electronic-structure uncertainty can most readily alter the interpretation, these two representative adsorption motifs were subjected to an explicit higher-level DFT benchmark. Two nested local models, Ti_4_O_8_ and Ti_6_O_12_, were extracted directly from the fully relaxed DFTB geometries around the H_2_ adsorption region; the corresponding Mg-containing models retained the surface-bound Mg species. No atomic coordinates were rebuilt before the single-point benchmark calculations.

Single-point calculations were performed at the PBE-D3(BJ)/def2-SVP level using Gaussian 16.^22–25^ Interaction energies were corrected for basis-set superposition error using the Boys–Bernardi counterpoise procedure,^26^ with the oxide fragment (including Mg where present) and H_2_ treated as the two fragments. The pristine Ti_4_O_8_ and Ti_6_O_12_ local models were treated as neutral singlets, whereas the corresponding Mg-containing models were treated as +2 singlets; H_2_ was treated as a neutral singlet. No artificial boundary passivation was introduced, and the same truncated oxide boundary was retained in the complex and fragment calculations. The two nested cluster sizes were used to assess the sensitivity of the local interaction to the truncation boundary.

To probe whether the DFTB adsorption geometry remained locally meaningful at the higher level of theory, fixed-framework H_2_ displacement scans were also performed for the larger Ti_6_O_12_ models. The Ti/O framework (and Mg in the modified model) was held at the DFTB geometry while H_2_ was displaced along the adsorbate-site direction. These calculations are used as targeted validation of the local adsorption motif and interaction sign; they are not interpreted as a quantitative replacement for a full 269-atom DFT nanoparticle calculation.

### 2.2 Adsorption energetics and electronic descriptors

#### 2.2.1 Adsorption energy and thermodynamic surrogates

For a single H_2_ molecule interacting with a nanoparticle, the electronic adsorption energy was defined as

1 *E*_ads_ = *E*(NP + H_2_) − *E*(NP) − *E*(H_2_),

where NP denotes the pristine or AE-modified nanoparticle. Negative values of *E*_ads_ correspond to exothermic adsorption, and all energies are reported per H_2_ molecule (1 eV = 96.485 kJ mol^−1^).

Because *E*_ads_ is a static electronic adsorption energy that omits finite-temperature contributions, we further constructed finite-temperature-corrected adsorption enthalpy surrogates,

2 Δ*H*_corr_ = *E*_ads_ + *δ*_corr_,

where *δ*_corr_ is a zero-point-and-thermal enthalpy correction, not an entropy term. We evaluated two limiting values at 298 K: a deciding lower bound of +6 kJ mol^−1^, appropriate for undissociated molecular or activated-molecular adsorption in which H_2_ remains intact and the correction is dominated by the loss of gas-phase translational and rotational enthalpy (∼(7/2)*RT*) together with a small change in zero-point energy; and an extreme upper bound of +25.96 kJ mol^−1^, of order the full gas-phase H_2_ zero-point vibrational energy (∼26 kJ mol^−1^), representing near-complete perturbation of the H–H stretch. Both the zero-point energy and the standard molar entropy of H_2_ that enters the free-energy expressions below are taken from standard gas-phase thermochemical data.^4^ The two values bracket the zero-point/thermal correction, so the resulting thermodynamic classifications are interpreted as sensitivity-bounded screening outcomes rather than unique quantitative predictions. Crucially, the entropy of adsorption is accounted for separately and enters only once, through the finite-temperature free-energy and desorption-temperature expressions below, so it is not double-counted in Δ*H*_corr_.

To connect adsorption energetics with near-ambient operability, the desorption midpoint temperature *T*_50_ was estimated within a Langmuir isotherm framework^27^ as3
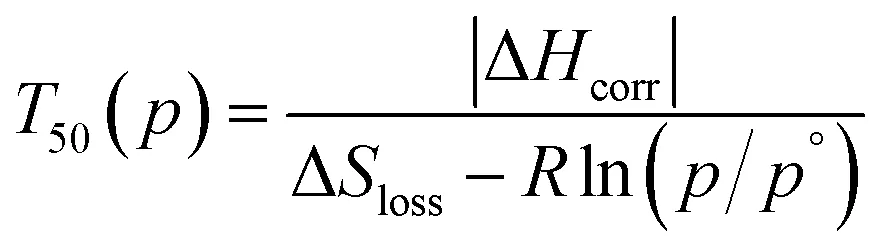
and is reported at *p* = 1 bar together with the corresponding equilibrium fractional coverage *θ*(298 K), where *p*° = 1 bar. To avoid ambiguity between electronic and thermodynamic screening criteria, descriptor-specific target windows were used throughout the analysis. The raw electronic adsorption energy *E*_ads_ was compared with the broad electronic screening window commonly used for reversible H_2_ binding, |*E*_ads_| = 0.2–0.6 eV, corresponding to −0.6 to −0.2 eV for exothermic adsorption. In contrast, the DOE/HyMARC-inspired thermodynamic target was applied only to the finite-temperature-corrected adsorption enthalpy, Δ*H*_corr_, with a target magnitude of 15–25 kJ mol^−1^ per H_2_, or −25 ≤ Δ*H*_corr_ ≤ −15 kJ mol^−1^ for exothermic adsorption. The practical desorption midpoint-temperature window was fixed as *T*_50_ = 250–400 K at 1 bar. A consistent entropy-loss assumption, Δ*S*_loss_ ≈ 8*R*, was used when mapping Δ*H*_corr_ onto the finite-temperature descriptors. With this entropy penalty, the 15–25 kJ mol^−1^ enthalpy target maps to *T*_50_ = 226–376 K at 1 bar, overlapping the practical *T*_50_ = 250–400 K operating window. Because *T*_50_ and Δ*G*° are obtained directly from Δ*H*_corr_, they are treated as derived thermodynamic diagnostics rather than statistically independent screening gates. Complete derivations of the free-energy, coverage, and midpoint-temperature expressions, together with the underlying assumptions, are provided in the SI.

#### 2.2.2 Frontier-orbital and conceptual-DFT descriptors

From the converged DFTB electronic structure, the frontier orbital energies *ε*_HOMO_ and *ε*_LUMO_ were extracted, together with the one-electron gap defined as *E*_gap_ = *ε*_LUMO_ − *ε*_HOMO_. Koopmans-like approximations were then used to estimate the vertical ionization potential and electron affinity as IP ≈ −*ε*_HOMO_ and EA ≈ −*ε*_LUMO_, respectively.^28^ On this basis, the Mulliken electronegativity *χ*, absolute hardness *η*, and Parr electrophilicity index *ω* were evaluated.^29,30^ These descriptors are used here comparatively, *i.e.*, to assess system-to-system trends across the studied series, rather than as quantitatively rigorous absolute observables. Their formal definitions are provided in the SI.

### 2.3 LLM-assisted screening framework

Recent studies have explored LLM-based and agentic workflows for scientific and materials-chemistry tasks, motivating the use of structured LLM assistance as an interpretive layer rather than as a predictive model.^31–33^

The GATOR framework (Fig. 1) was used as a structured, physics-guided workflow for screening H_2_ adsorption behavior across the studied systems. In the first stage, the DFTB+-derived descriptors were evaluated through a deterministic screening sequence including adsorption mode, binding regime, the broad raw-electronic *E*_ads_ window, and the DOE/HyMARC finite-temperature-corrected adsorption-enthalpy window. The *T*_50_ and Δ*G*° descriptors were then used as thermodynamic diagnostics derived from Δ*H*_corr_, rather than as statistically independent gates. The rule-based procedure combined the primary thermodynamic classification with the derived diagnostic descriptors to assign systems into PASS, BORDERLINE, or FAIL categories. Only borderline systems were forwarded to the retrieval stage, whereas clearly non-viable systems retained their thermodynamic classification without additional literature augmentation.

**Fig. 1:**
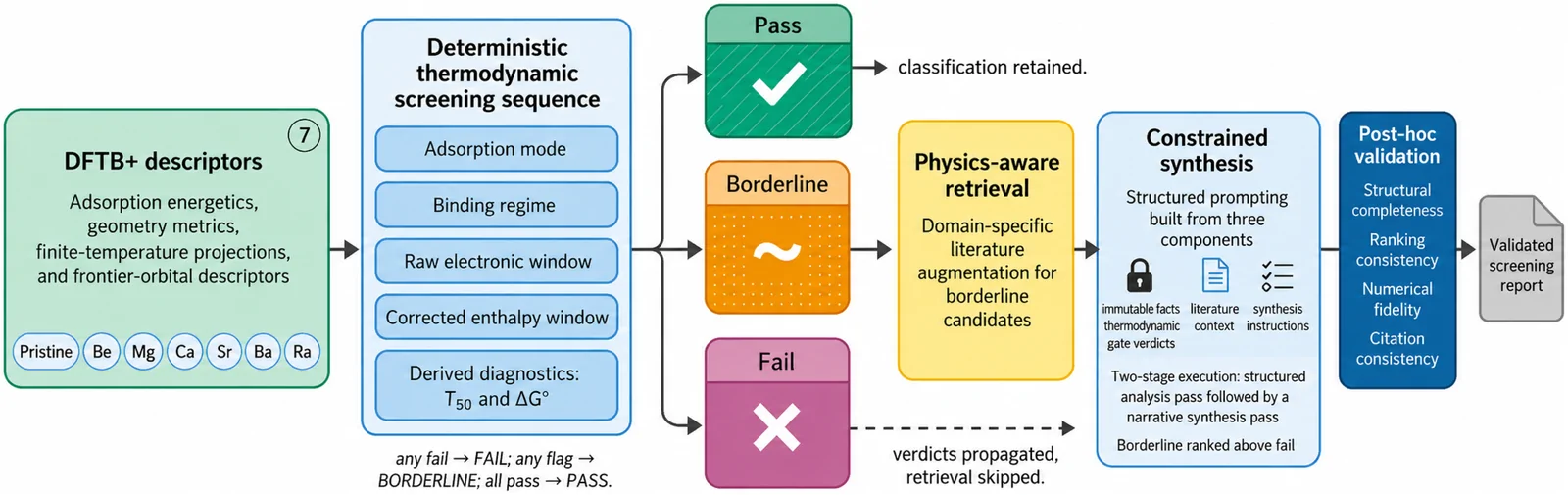
GATOR (Gate-Augmented Thermodynamic Ordering and Retrieval) framework for physics-guided screening of H_2_ adsorption on alkaline-earth-modified TiO_2_ nanoparticles. The workflow combines DFTB-derived descriptors with a deterministic thermodynamic screening sequence comprising adsorption mode, binding regime, a broad raw-electronic screening window, and finite-temperature-corrected enthalpy, while *T*_50_ and Δ*G*° are used as derived diagnostic descriptors for interpreting uptake-release behavior. Only borderline systems proceed to retrieval; fail systems retain their classification. A constrained large language model is then used for structured synthesis under fixed gate constraints, followed by post-hoc validation of ranking consistency, numerical fidelity, and citation consistency.

In the second stage, a constrained large language model was applied to a structured prompt containing the fixed gate outputs, retrieved literature context where applicable, and synthesis instructions. It was used to organize comparative analysis, ranking, and follow-up suggestions while preserving the hierarchy imposed by the physics-based screening layer. A post-hoc validation step was then used to verify structural completeness, ranking consistency (no failed system may be ranked above a non-failed one, so the language model cannot override the gate verdicts), numerical agreement within a 1% tolerance of the source descriptors, and citation consistency. In this way, the workflow preserves physically grounded decision-making while providing an additional layer of structured interpretation. All reported analyses used three independent models (Claude Opus 4.6, Gemini 3.1 Pro, and GPT-5.4) at a decoding temperature of 0.2 with five runs per model, and all fifteen runs passed all four validation layers; the exact prompt templates, the gate-configuration file, and the per-run provenance are reproduced in SI Section S2.3.

## 3 Results and discussion

### 3.1 Structural and energetic analysis of H_2_ adsorption

Fig. 2 shows the relaxed pristine and AE-modified TiO_2_ nanoparticles together with their representative relaxed H_2_-adsorbed geometries. Table 1 summarizes the intramolecular H–H bond length *r*_H–H_, the shortest metal–hydrogen separation *r*_H–AE_, and the local anchoring distance *R*_O–AE_, while the underlying DFTB total energies used to derive the adsorption energies are listed in Table S1. Using the conventional structural thresholds for molecular (*r*_H–H_ < 0.80 Å), activated (0.80–0.95 Å), and dissociative (*r*_H–H_ > 0.95 Å) adsorption, a clear hierarchy of H_2_ interaction strengths emerges among the representative configurations examined. Although the TiO_2_ backbones in Fig. 2 appear very similar at the full-particle scale, quantitative comparison confirms that this similarity reflects preservation of the parent oxide framework rather than an imposed geometrical constraint. Across the six bare AE-modified nanoparticles, the 269-atom Ti/O framework exhibits an aligned RMSD of only 0.052–0.055 Å relative to pristine TiO_2_, with maximum individual Ti/O displacements of 0.135–0.204 Å. The perturbation is more pronounced locally: within 4.0 Å of the anchoring surface O atom, the framework RMSD increases to 0.102–0.154 Å (Table S2). Thus, adsorption of a single AE adatom produces a localized surface relaxation rather than a global reconstruction of the approximately 1.8 nm oxide scaffold.

**Fig. 2:**
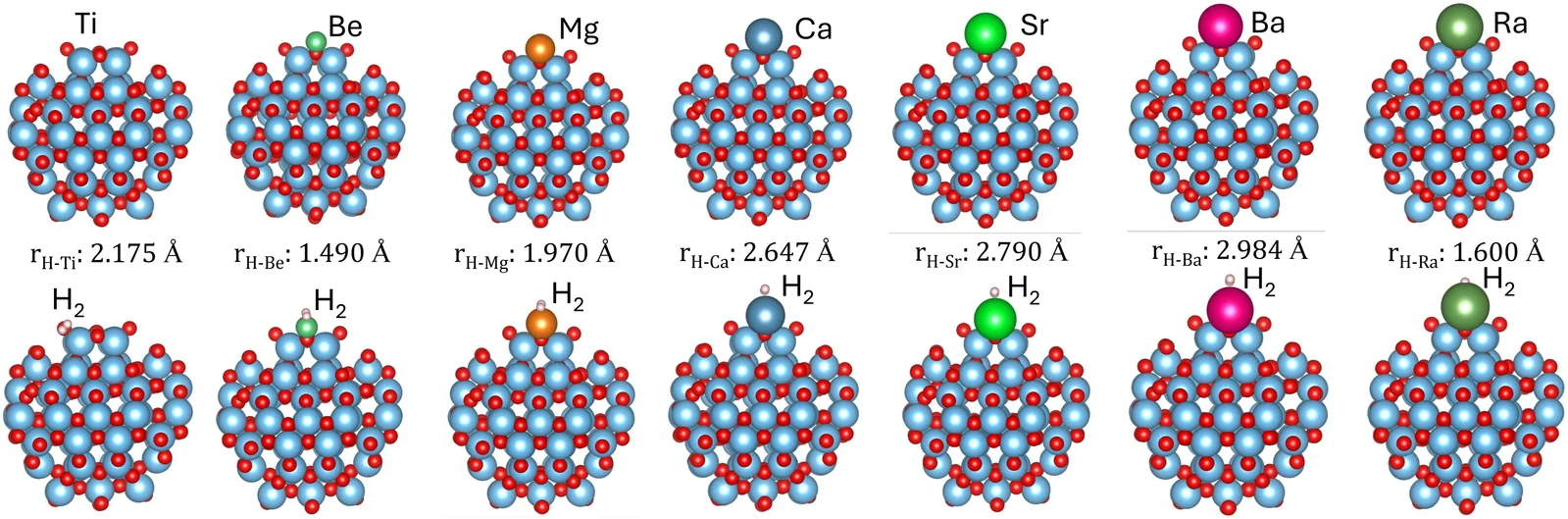
Optimized structures of pristine and alkaline-earth-modified TiO_2_ nanoparticles before and after H_2_ adsorption. The top row shows the relaxed nanoparticles prior to H_2_ adsorption; for the modified systems, the alkaline-earth atom (AE = Be, Mg, Ca, Sr, Ba, Ra) is highlighted as the larger colored sphere, whereas the pristine panel shows the unmodified TiO_2_ nanoparticle. The bottom row shows the representative relaxed H_2_-adsorbed geometries, with one H_2_ molecule initially placed above the AE-centered adsorption region, or near a representative surface Ti site for pristine TiO_2_. The values beneath the top panels indicate the shortest H–M distance in the adsorbed state: pristine (H–Ti) 2.176 Å, Be 1.491 Å, Mg 1.971 Å, Ca 2.647 Å, Sr 2.791 Å, Ba 2.985 Å, and Ra 1.600 Å. The same viewing direction and particle scale are used for all systems to facilitate direct comparison; consequently, localized relaxations around the AE adsorption site are visually compressed in the full-particle representation. The apparent similarity of the TiO_2_ backbone should therefore not be interpreted as identical optimized coordinates. Quantitative framework and local displacement metrics are reported in Table S2, while the distinct local H–H, H–M, and O–AE distances are reported in Table 1. The AE species are surface-bound adatoms and do not substitute Ti or O atoms in the nanoparticle framework. Ti atoms are shown in light blue, O atoms in red, AE atoms in the indicated colors, and H_2_ is shown only in the bottom row.

**Table 1:** H_2_ bond activation and nearest-neighbor distances on pristine and alkaline-earth (AE)-modified TiO_2_ nanoparticles

System	*r*_H–H_ (Å)	*r*_H–AE_ (Å)^a^	*R*_O–AE_ (Å)^b^	Elong (%)^c^	**Mode** d
Pristine^e^	0.773	2.176	—	4.33	M
Be–TiO_2_	0.823	1.491	1.354	11.08	**A**
Mg–TiO_2_	0.778	1.971	1.672	4.97	M
Ca–TiO_2_	0.760	2.647	2.479	2.51	M
Sr–TiO_2_	0.762	2.791	2.627	2.81	M
Ba–TiO_2_	0.760	2.985	2.805	2.51	M
Ra–TiO_2_	0.751	1.600	2.508	1.35	M

a Shortest H–AE distance.

b Nearest surface O–AE distance.

c 100 (*r*_H–H_ − *r*_0_)/*r*_0_, with *r*_0_ = 0.741 Å.

d M: molecular (<0.80 Å); A: activated (0.80–0.95 Å); D: dissociative (>0.95 Å).

e Pristine TiO_2_; *r*_H–AE_ is the closest H-surface-atom distance.

Likewise, the H_2_ interaction geometries are not identical after relaxation. Although analogous initial placements were deliberately used to provide a controlled comparison, all coordinates were subsequently optimized without positional or symmetry constraints. The resulting H–AE separations span 1.491–2.985 Å, the O–AE anchoring distances span 1.354–2.805 Å, and the H–H bond lengths vary from 0.751 to 0.823 Å across the AE series (Table 1). The visually similar global orientations in Fig. 2 therefore mask substantial differences in the local adsorption environments that are directly reflected in the adsorption-energy trends.

Be–TiO_2_ exhibits the strongest interaction, with an elongated H–H bond (*r*_H–H_ = 0.823 Å; activated regime), the shortest H–AE contact (1.491 Å), and the most exothermic adsorption energy (*E*_ads_ = −1.565 eV). These features place Be–TiO_2_ in a distinct strong-binding regime, although the absolute magnitude of the adsorption energy should be interpreted at the present screening level. At the opposite end of the series, Ca–, Sr–, and Ba–TiO_2_ retain largely molecular H_2_ (*r*_H–H_ ≈ 0.76 Å), display long H–AE separations (∼2.65–2.98 Å), and show only mildly exothermic adsorption energies (−0.18 to −0.21 eV). Their comparatively large *R*_O–AE_ values are consistent with weaker, predominantly physisorptive interactions and limited H_2_ activation.

Between these two extremes, Mg–TiO_2_ shows intermediate behavior, with modest H–H elongation (*r*_H–H_ = 0.778 Å; molecular adsorption), a relatively short H–Mg contact (1.971 Å), and an adsorption energy of −0.551 eV. Within the present DFTB-based representative-configuration screening picture, Mg–TiO_2_ and pristine TiO_2_ constitute the two intermediate-binding reference cases. Pristine TiO_2_ exhibits a comparable DFTB-level interaction, with *r*_H–H_ = 0.773 Å and *E*_ads_ = −0.496 eV.

Ra–TiO_2_ is an instructive outlier. Despite a relatively strong adsorption energy (*E*_ads_ = −0.689 eV) and a short H–Ra distance (1.600 Å), the H–H bond remains close to its molecular value (*r*_H–H_ = 0.751 Å). This suggests a predominantly polarization-driven molecular interaction with limited occupation of the H_2_σ* orbital, indicating that stronger adsorption does not necessarily imply stronger H–H activation.

Overall, shorter local H–M contacts correlate with more exothermic adsorption and, in the limiting case of Be–TiO_2_, with clear H_2_ activation. In contrast, systems with long H–AE distances and large O–AE separations, particularly Ca–, Sr–, and Ba–TiO_2_, remain molecular and weakly bound. Taken together, these local structural metrics are consistent with the energetic trends and place Mg–TiO_2_ and pristine TiO_2_ in an intermediate-binding regime, while the quantitative framework analysis shows that AE-induced structural relaxation remains predominantly localized rather than involving wholesale reconstruction of the TiO_2_ nanoparticle.

### 3.2 Higher-level DFT validation of representative adsorption motifs

The higher-level calculations provide an important qualification of the DFTB screening results. At the DFTB-derived adsorption geometries, the counterpoise-corrected PBE-D3(BJ)/def2-SVP interaction energies for pristine TiO_2_ are −0.313 and −0.350 eV for the Ti_4_O_8_ and Ti_6_O_12_ local models, respectively. The corresponding values for Mg–TiO_2_ are −0.173 and −0.138 eV. Thus, both nested cluster sizes retain an attractive H_2_ interaction for both representative systems, and changing the local-model size modifies the interaction energy by only ∼0.04 eV within each series. Full numerical benchmark and displacement-scan data are provided in Tables S4 and S5 of the SI.

Importantly, however, the higher-level calculations do not reproduce the relative pristine/Mg energetic ordering obtained from the full-nanoparticle DFTB calculations. DFTB gives *E*_ads_ = −0.496 eV for pristine TiO_2_ and −0.551 eV for Mg–TiO_2_, whereas the larger Ti_6_O_12_ PBE-D3(BJ) models yield local counterpoise-corrected interaction energies of −0.350 and −0.138 eV, respectively. Consequently, the small DFTB energetic preference for Mg over pristine TiO_2_ is not method-independent. We therefore do not interpret the DFTB pristine/Mg ordering as a quantitatively validated energetic ranking; rather, the DFTB values are retained as internally consistent screening descriptors for the complete AE series.

The higher-level distance scans further probe the local structural stability of the two representative adsorption motifs while revealing method-dependent adsorbate-site separations. For pristine TiO_2_, the DFTB H_2_-center-Ti separation is approximately 2.15 Å. The raw PBE-D3(BJ) interaction profile exhibits a shallow local minimum near this distance, while the counterpoise-corrected interactions remain attractive throughout the sampled 1.95–2.35 Å interval. For Mg–TiO_2_, the DFTB H_2_-center-Mg separation is approximately 1.94 Å, whereas both the raw and counterpoise-corrected PBE-D3(BJ) profiles bracket a local minimum at approximately 2.34 Å between the sampled 2.14 and 2.54 Å points. Thus, higher-level DFT supports an attractive Mg–H_2_ adsorption motif but predicts a longer preferred local separation by approximately 0.4 Å. Taken together, the validation calculations support the qualitative existence of both representative adsorption configurations while demonstrating that their relative energetic ranking and, particularly for Mg, the precise adsorbate-surface distance are sensitive to the electronic-structure method.

### 3.3 Thermodynamic screening and deliverability assessment

To connect the structural trends with practical operability, Table 2 reports finite-temperature-corrected adsorption enthalpies obtained under two limiting correction schemes, while Table 3 lists the corresponding desorption midpoint temperatures *T*_50_ and standard-state free energies Δ*G*°(298 K).

**Table 2:** Finite-temperature-corrected H_2_ adsorption enthalpies at 298 K (kJ mol^−1^) with DOE screening classification. Values are DFTB+ screening descriptors within the same PTBP-based model chemistry and are intended for relative trend comparison, not as high-precision absolute adsorption enthalpies

System	Δ*H*_+6_ (kJ mol^−1^)^a^	**DOE** ^c^	Δ*H*_+25.96_ (kJ mol^−1^)^b^	**DOE** c
Pristine TiO_2_	−41.84	Strong	−21.88	**Inside**
Be–TiO_2_	−145.00	Strong	−125.04	Strong
Mg–TiO_2_	−47.16	Strong	−27.20	Strong
Ca–TiO_2_	−13.80	Weak	6.16	Endo
Sr–TiO_2_	−13.62	Weak	6.34	Endo
Ba–TiO_2_	−11.44	Weak	8.52	Endo
Ra–TiO_2_	−60.45	Strong	−40.49	Strong

a Δ*H*_+6_ = Δ*E*_ads_ + 6 kJ mol^−1^, a lower-bound zero-point-and-thermal enthalpy correction per H_2_ at 298 K (molecular physisorption; the deciding scheme).

b Δ*H*_+25.96_ = Δ*E*_ads_ + 25.96 kJ mol^−1^, an extreme upper-bound correction of order the full gas-phase H_2_ zero-point energy (∼26 kJ mol^−1^), reported only as a sensitivity.

c DOE/HyMARC adsorption-enthalpy window (applied to the finite-temperature-corrected enthalpy Δ*H*_corr_, not to raw *E*_ads_): −25 ≤ Δ*H* ≤ −15 kJ mol^−1^ (“Inside”). Strong: Δ*H* < − 25 (too exothermic); weak: −15 < Δ*H* < 0 (too shallow); endo.: Δ*H* > 0.

**Table 3:** Langmuir-model desorption midpoint temperatures at 1 and 30 bar^a^ and standard-state free energies of H_2_ adsorption at 298 K^b^

System	*T* ^1 bar^_50_ (K)	*T* ^30 bar^_50_ (K)	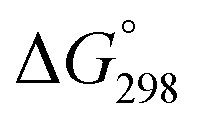 (kJ mol^−1^)
Pristine TiO_2_	629	1094	−22.0
Be–TiO_2_	2180	3792	−125.2
Mg–TiO_2_	709	1233	−27.3
Ca–TiO_2_	207	361	6.0
Sr–TiO_2_	205	356	6.2
Ba–TiO_2_	172	299	8.4
Ra–TiO_2_	909	1581	−40.6

a Temperature at which half of the H_2_ monolayer remains adsorbed under the Langmuir isotherm at the indicated pressure; higher *T*_50_ indicates stronger binding and more difficult desorption. The values were evaluated using Δ*S*_loss_ ≈ 8*R* and *T*_50_(*p*) = |Δ*H*_corr_|/[Δ*S*_loss_ − *R* ln(*p*/*p*°)].

b
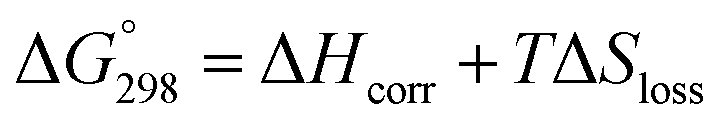
 at 298 K, where Δ*H*_corr_ is negative for exothermic adsorption and Δ*S*_loss_ is treated as a positive entropy penalty; negative values indicate spontaneous adsorption at standard state.

Following the descriptor-specific screening windows defined in Section 2.2, Fig. 3 compares the raw electronic adsorption energy *E*_ads_ with the broad electronic screening window commonly used for near-ambient reversible H_2_ binding (|*E*_ads_| = 0.2–0.6 eV).^34–37^ The plotted Δ*H*_corr_ values are lower-bound finite-temperature surrogates, screened separately against the DOE/HyMARC enthalpy window (Table 2). Within this comparison, pristine TiO_2_ and Mg–TiO_2_ lie inside the electronic window, Ca–, Sr–, and Ba–TiO_2_ cluster near or below the weak-binding boundary, and Be– and Ra–TiO_2_ fall on the strong-binding side. Fig. 4 extends this analysis through a multi-criteria screening panel based on |*E*_ads_|, |Δ*H*_corr_^(+6)^|, and *T*_50_, with each bar judged against its own screening window.

**Fig. 3:**
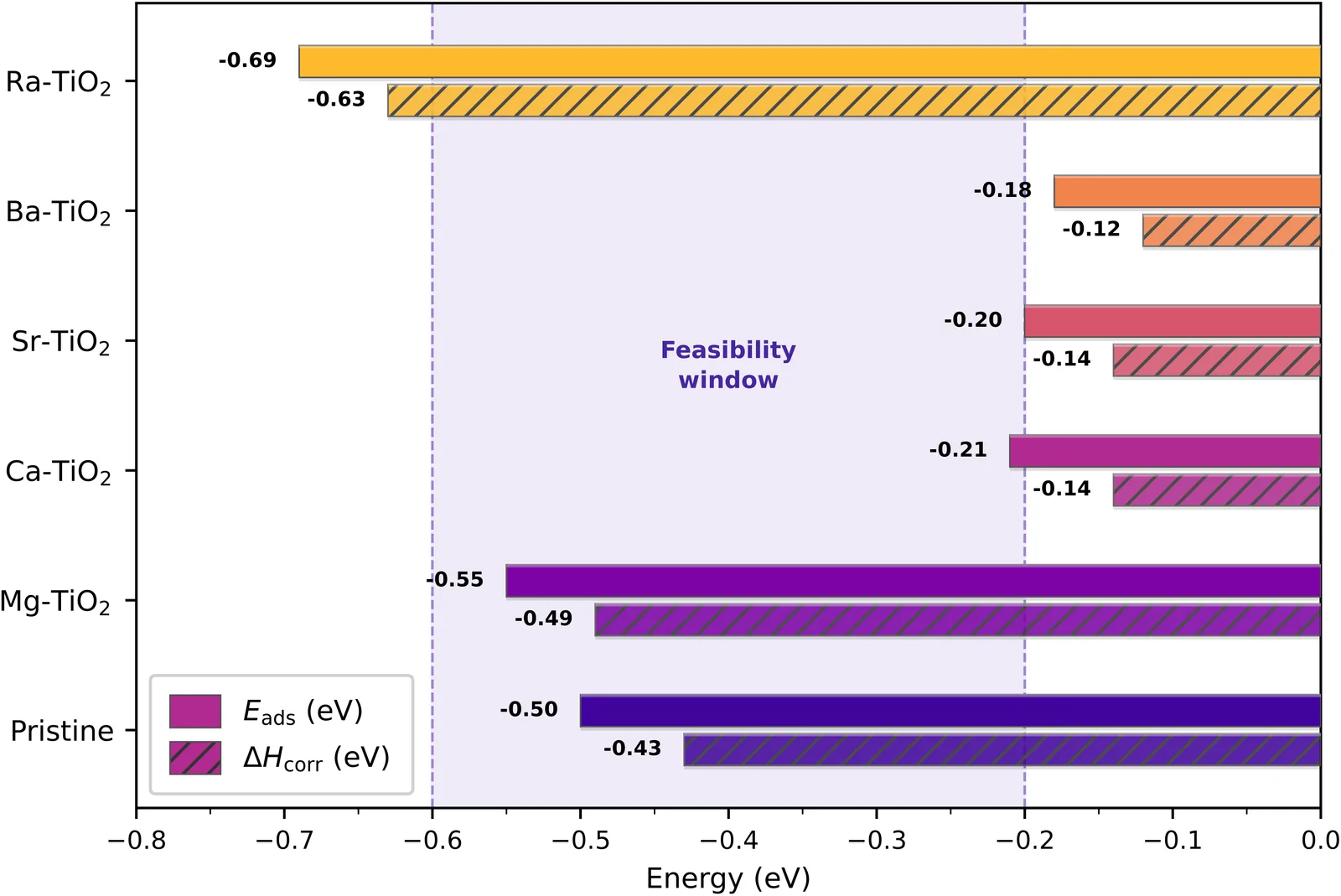
Screening of H_2_ adsorption on pristine and alkaline-earth-modified TiO_2_ nanoparticles against the broad raw electronic adsorption-energy window. The bars show the electronic adsorption energy *E*_ads_ (blue) and a lower-bound finite-temperature enthalpy surrogate Δ*H*_corr_ (orange), where Δ*H*_corr_ = *E*_ads_ + 6 kJ mol^−1^ per H_2_. This +6 correction is included only as a conservative lower-bound zero-point/thermal enthalpy-correction scenario; Table 2 additionally reports an extreme upper-bound correction of +25.96 kJ mol^−1^ per H_2_ (of order the gas-phase H_2_ zero-point energy), which modifies the thermodynamic classification of several borderline systems. The shaded band (−0.6 to −0.2 eV) denotes the broad raw electronic *E*_ads_ screening window, |*E*_ads_| = 0.2–0.6 eV. This electronic-energy window is distinct from the DOE/HyMARC-inspired adsorption-enthalpy window of 15–25 kJ mol^−1^ applied to the finite-temperature-corrected Δ*H*_corr_ in Table 2, and the two should not be treated as equivalent. Be–TiO_2_ is excluded from this figure due to anomalously strong binding (*E*_ads_ = −1.56 eV) and is discussed separately in the text.

**Fig. 4:**
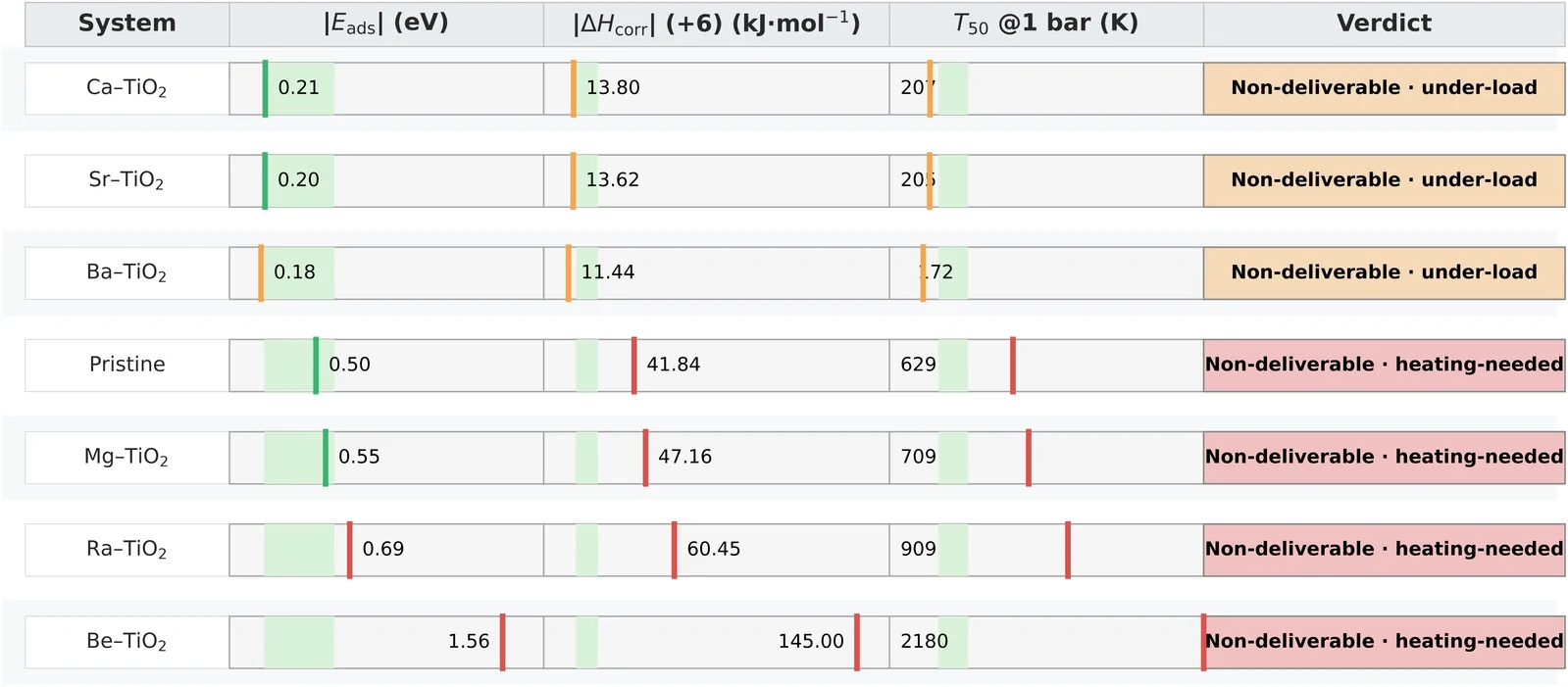
Multi-criteria screening panel for H_2_ adsorption on pristine and alkaline-earth-modified TiO_2_ nanoparticles at 298 K. From left to right, the bullet bars report |*E*_ads_|, |Δ*H*_corr_^(+6)^|, and *T*_50_ at 1 bar. The green bands indicate the descriptor-specific screening windows (|*E*_ads_| = 0.2–0.6 eV, |Δ*H*_corr_^(+6)^| = 15–25 kJ mol^−1^, and *T*_50_ = 250–400 K). Colored ticks denote the actual values for each system (green = within range, orange = under-load side, red = heating-needed side). Numerical values are printed inside the |*E*_ads_| and |Δ*H*_corr_^(+6)^| bars, whereas *T*_50_ values are shown to the left of the corresponding bars. The rightmost panel summarizes the thermodynamic screening classification based on the combined behavior of *E*_ads_, Δ*H*_corr_, 
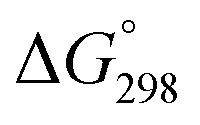
, and *T*_50_. Under the entropy-consistent *T*_50_ calculation, pristine TiO_2_ and Mg–TiO_2_ move to the stronger-binding/heating-needed side of the desorption window, although they remain the most balanced systems within the present series. The *T*_50_ values were evaluated using the same entropy-loss convention, Δ*S*_loss_ ≈ 8*R*, used for the free-energy analysis.

Table 4 shows that the absolute *T*_50_-based deliverability verdict is sensitive to the selected Δ*S*_loss_ and *δ*_corr_ constants. For example, using Δ*S*_loss_ = 15.7*R* with *δ*_corr_ = +6 kJ mol^−1^ shifts pristine TiO_2_ and Mg–TiO_2_ into the practical *T*_50_ window. Therefore, the deliverability label should be interpreted as convention-dependent within this screening model. However, because the same correction and entropy assumptions are applied consistently to both systems, the relative ordering within the DFTB-derived thermodynamic projection is unchanged: Mg–TiO_2_ remains slightly stronger-binding than pristine TiO_2_ under every tested combination. This DFTB-level ordering should not be interpreted as method-independent, as demonstrated by the higher-level benchmark in Section 3.2.

**Table 4:** Sensitivity of the desorption midpoint temperature to the selected entropy-loss and enthalpy-correction constants for the two borderline systems. The midpoint temperature was evaluated at 1 bar using *T*_50_ = |Δ*H*_corr_|/Δ*S*_loss_. The practical window is *T*_50_ = 250–400 K

System	*δ*_corr_ (kJ mol^−1^)	Δ*S*_loss_	Δ*H*_corr_ (kJ mol^−1^)	*T*_50_ (K)
Pristine TiO_2_	+6.00	8*R*	−41.84	629
Pristine TiO_2_	+6.00	12*R*	−41.84	419
Pristine TiO_2_	+6.00	15.7*R*	−41.84	321
Pristine TiO_2_	+25.96	8*R*	−21.88	329
Pristine TiO_2_	+25.96	12*R*	−21.88	219
Pristine TiO_2_	+25.96	15.7*R*	−21.88	168
Mg–TiO_2_	+6.00	8*R*	−47.16	709
Mg–TiO_2_	+6.00	12*R*	−47.16	473
Mg–TiO_2_	+6.00	15.7*R*	−47.16	361
Mg–TiO_2_	+25.96	8*R*	−27.20	409
Mg–TiO_2_	+25.96	12*R*	−27.20	273
Mg–TiO_2_	+25.96	15.7*R*	−27.20	208

Across these windows, three thermodynamic regimes are observed. Pristine TiO_2_ and Mg–TiO_2_ remain within the broad raw electronic adsorption-energy window; however, their finite-temperature-corrected enthalpies, |Δ*H*_corr_^(+6)^| = 41.84 and 47.16 kJ mol^−1^, are stronger than the 15–25 kJ mol^−1^ target. Their corresponding desorption midpoint temperatures are *T*_50_ = 629 and 709 K at 1 bar, respectively, placing both systems above the practical 250–400 K window. Therefore, they are not assigned as near-ambient deliverable systems, but represent the most balanced strong-binding cases in the present series. Ca–, Sr–, and Ba–TiO_2_ remain weak-binding systems, with *T*_50_ = 207, 205, and 172 K, respectively, indicating insufficient near-ambient retention. Ra–TiO_2_ and Be–TiO_2_ remain excessive-binding systems, with *T*_50_ = 909 and 2180 K, respectively, and require heating-assisted desorption. In this framework, Δ*H*_corr_ is the primary thermodynamic descriptor, whereas *T*_50_ and 
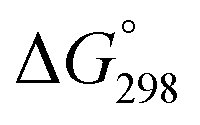
 are derived diagnostic quantities.

This thermodynamic ranking is fully consistent with the structural indicators in Table 1. Systems with pronounced H–H elongation, most notably Be–TiO_2_, occupy the strong-binding regime, whereas weakly perturbed H–H distances in Ca–, Sr–, and Ba–TiO_2_ correlate with under-loaded, predominantly physisorptive behavior. Pristine TiO_2_ and Mg–TiO_2_ remain intermediate between these two extremes and therefore occupy the most balanced part of the adsorption landscape within the present representative single-site model. Taken together, the decision panel identifies pristine TiO_2_ and Mg–TiO_2_ as the two most balanced adsorption cases within the present DFTB-based screening model, but the entropy-consistent thermodynamic descriptors place both systems on the stronger-binding side of the ideal reversible-storage window. The complete gate-by-gate verdicts for all seven systems are reported in SI Table S6. Fig. S1 visualizes the same thermodynamic data as a coverage heatmap and reproduces the separation between weak-binding, stronger-binding, and excessive-binding regimes. Fig. 5 translates the free-energy data into pressure-dependent isotherms. Three behaviors emerge: (i) Be–TiO_2_ and Ra–TiO_2_ reach *θ* ≈ 1 already at ∼10^−2^–10^−1^ bar, loading readily but risking poor deliverability; (ii) pristine TiO_2_ and Mg–TiO_2_ reach high coverages by ∼1–10 bar, consistent with stronger-than-ideal adsorption and limited desorption ease; (iii) Ca/Sr/Ba–TiO_2_ remain sparsely populated at ≤1 bar, requiring higher charging pressures. Lateral adsorbate interactions at finite coverage would shift the projected *θ*(*p*, *T*) and *T*_50_ relative to the ideal Langmuir limit, with attractive interactions stabilizing adsorption and repulsive interactions reducing apparent loading.

**Fig. 5:**
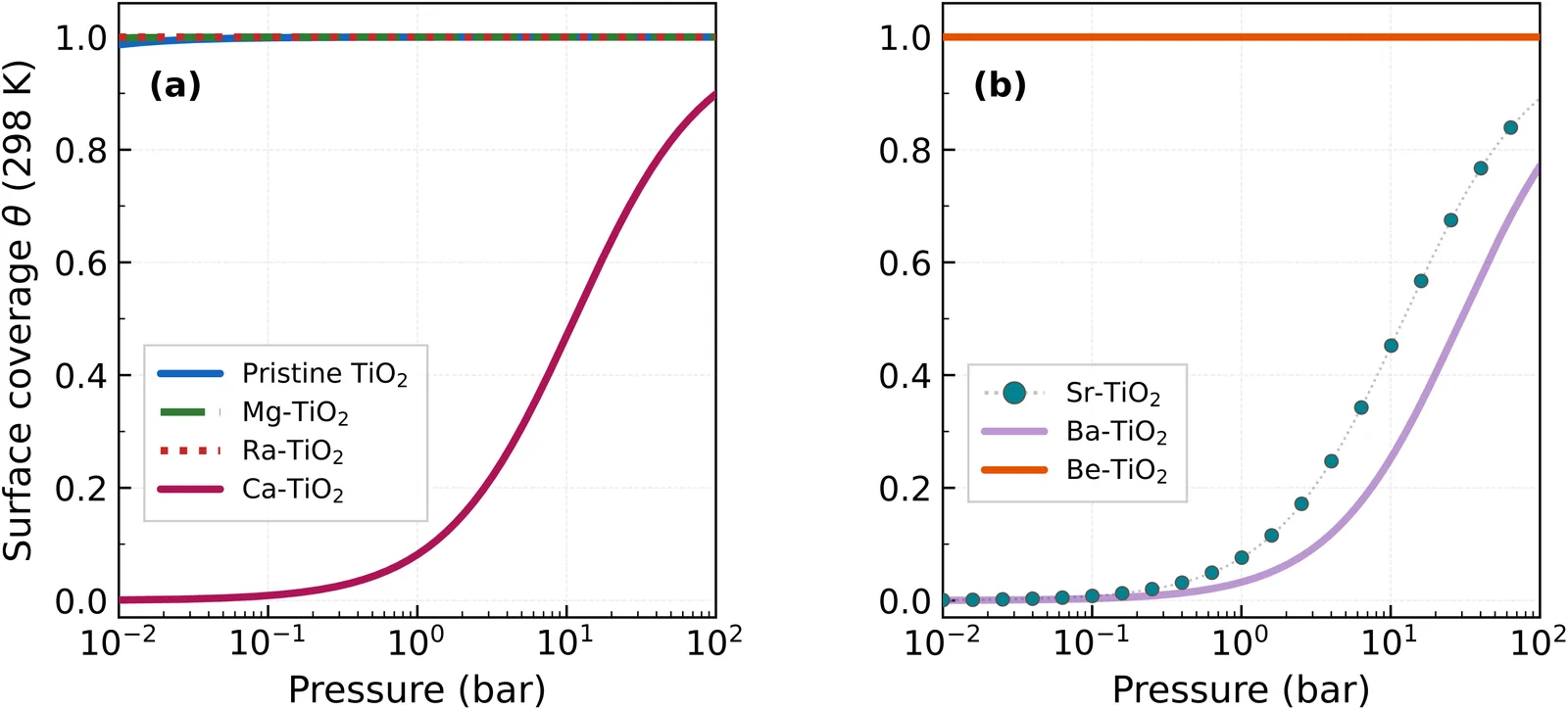
Langmuir coverage *vs.* pressure at 298 K for pristine and AE–TiO_2_ nanoparticles. Curves were computed from Δ*G*°(298 K) in Table 3. Panel (a) shows pristine TiO_2_, Mg–TiO_2_, Ca–TiO_2_, and Ra–TiO_2_; panel (b) shows Sr–TiO_2_, Ba–TiO_2_, and Be–TiO_2_. Assumes identical, non-interacting adsorption sites and ideal-gas H_2_. Site heterogeneity and lateral adsorbate interactions are not captured and could broaden or shift the practical adsorption response.

### 3.4 Electronic structure descriptors

As a complementary diagnostic, frontier-orbital and conceptual-DFT descriptors were evaluated for both the bare and H_2_-adsorbed clusters. Across the full series, the HOMO–LUMO gap remains nearly constant at *E*_g_ ≈ 3.58–3.59 eV, consistent with the quantum-confinement-induced widening expected for ∼1.8 nm anatase crystallites,^38^ and changes by less than 0.02 eV upon AE modification or H_2_ adsorption. Likewise, the Mulliken electronegativity (*χ* ≈ 6.32 eV), absolute hardness (*η* ≈ 1.79–1.80 eV), and electrophilicity index (*ω* ≈ 11.1 eV) vary only in the second decimal place across all systems.^29,30^ The near-invariance of these global descriptors indicates that the differences in H_2_ adsorption behavior are governed primarily by local adsorption geometry and local charge redistribution rather than by major global electronic-structure reorganization of the nanoparticle. Even the strongest-binding case, Be–TiO_2_, shows only negligible change in *E*_g_, underscoring that adsorption strength and global gap renormalization can remain effectively decoupled for closed-shell adsorbates on wide-gap oxide nanoparticles at this coverage. Complete numerical values, together with the H_2_-induced shifts in *E*_g_, *χ*, and *η*, are reported in the SI (Table S3 and Fig. S2).

Within the DFTB-based screening framework, the identification of pristine TiO_2_ and Mg–TiO_2_ as the most balanced systems in the present series is consistent with previous experimental and computational studies in which Mg modification tempers the surface reactivity of TiO_2_ without driving it into the extreme chemisorptive regime observed for Be–TiO_2_.^10,11^ These results should therefore be interpreted as site-specific thermodynamic trends for a fixed anatase nanoparticle model rather than as system-level storage-capacity benchmarks against porous sorbent materials. The overall agreement between the structural, energetic, and electronic descriptors supports pristine TiO_2_ and Mg–TiO_2_ as co-priority targets for higher-fidelity follow-up studies, including coverage-dependent, vibrational, and kinetic analyses.

### 3.5 LLM-assisted screening diagnostics

Fig. 6(a) places the computed |*E*_ads_| values relative to the broad raw electronic adsorption-energy window and resolves the same three thermodynamic regimes already identified above. Pristine TiO_2_ and Mg–TiO_2_ show intermediate adsorption strengths (∼48–53 kJ mol^−1^), which lie inside this electronic window; their classification as the most balanced strong-binding cases instead arises from the finite-temperature-corrected adsorption enthalpy, which falls on the strong-binding side of the 15–25 kJ mol^−1^ enthalpy window under the lower-bound correction, while their entropy-consistent *T*_50_ values place them on the stronger-binding side of the practical desorption window. This behavior is consistent with their intermediate structural character, in which H_2_ remains molecular but exhibits modest elongation and relatively short H–metal contacts. In contrast, Be–TiO_2_ and Ra–TiO_2_ occupy the strong-binding regime, with large |*E*_ads_| values and correspondingly high desorption temperatures, leading to limited practical reversibility under near-ambient conditions.

**Fig. 6:**
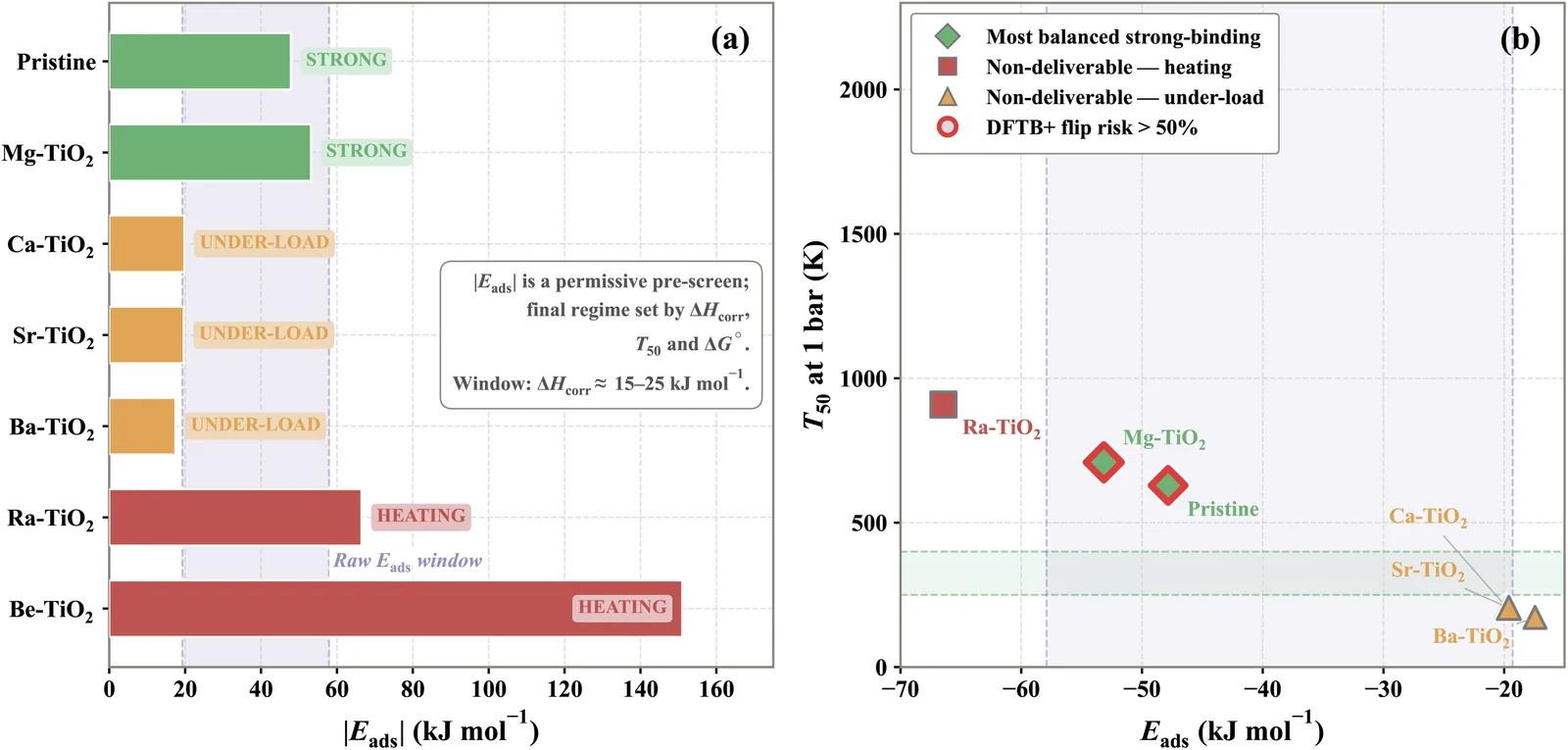
Physics-guided screening of H_2_ adsorption on AE-modified anatase TiO_2_ NPs with LLM-supported interpretation. (a) Thermodynamic screening of |*E*_ads_| against the broad raw electronic adsorption-energy window (purple band), with systems assigned to three regimes: weak-binding/under-load, electronically admissible and most balanced strong-binding, and strong-binding/heating-needed. Because this window is only a permissive pre-screen, the regime assignments are based on the finite-temperature-corrected enthalpy, *T*_50_, and Δ*G*°, rather than on |*E*_ads_| alone. (b) Two-descriptor screening map of *E*_ads_*versus* desorption midpoint temperature *T*_50_ at 1 bar. The green and purple bands indicate the practical *T*_50_ range and broad raw electronic *E*_ads_ window, respectively. Marker shape encodes the regime classification, marker size scales with the number of LLM anomaly flags, and red outlines identify systems with an LLM-predicted DFTB+ flip risk above 50%. Be–TiO_2_ is outside the plotted *E*_ads_ range in panel (b) because of its exceptionally strong adsorption and very high *T*_50_ value.

Fig. 6(a) also shows that Ca–, Sr–, and Ba–TiO_2_ lie near the weak-binding edge of the raw electronic adsorption-energy window while remaining thermodynamically unfavorable for practical adsorption at 298 K. Their positive Δ*G*° values and very low *T*_50_ values indicate negligible equilibrium coverage under ambient conditions. Taken together, these results show that enthalpy-based screening alone is insufficient and must be interpreted together with entropy and free-energy corrections when assessing practical deliverability.

Fig. 6(b) maps the same systems in the (*E*_ads_, *T*_50_) descriptor space, directly connecting adsorption energetics to desorption behavior. Pristine TiO_2_ and Mg–TiO_2_ remain electronically admissible but thermodynamically stronger than the ideal *T*_50_ window, while the strong-binding (Be–, Ra–) and weak-binding (Ca–, Sr–, Ba–) systems fall outside it at high and low *T*_50_, respectively. The additional LLM-based overlays in Fig. 6(b) provide a complementary uncertainty-oriented interpretation by associating larger anomaly counts with strong-binding systems and highlighting elevated DFTB+ flip risk for these most balanced strong-binding cases.

Fig. 7 provides a complementary summary of the vocabulary distributions from independent screening runs for two representative models (GPT-5.4 and Claude Opus 4.6); cross-model agreement across all three benchmarked models is quantified in Fig. S3. The two models converge on a shared physics-centered lexicon, with the differences in term prominence reflecting emphasis rather than disagreement—one model weighting correction- and sensitivity-related terms more strongly and the other highlighting anomaly detection and regime classification. This overall agreement supports the robustness of the screening conclusions.

**Fig. 7:**
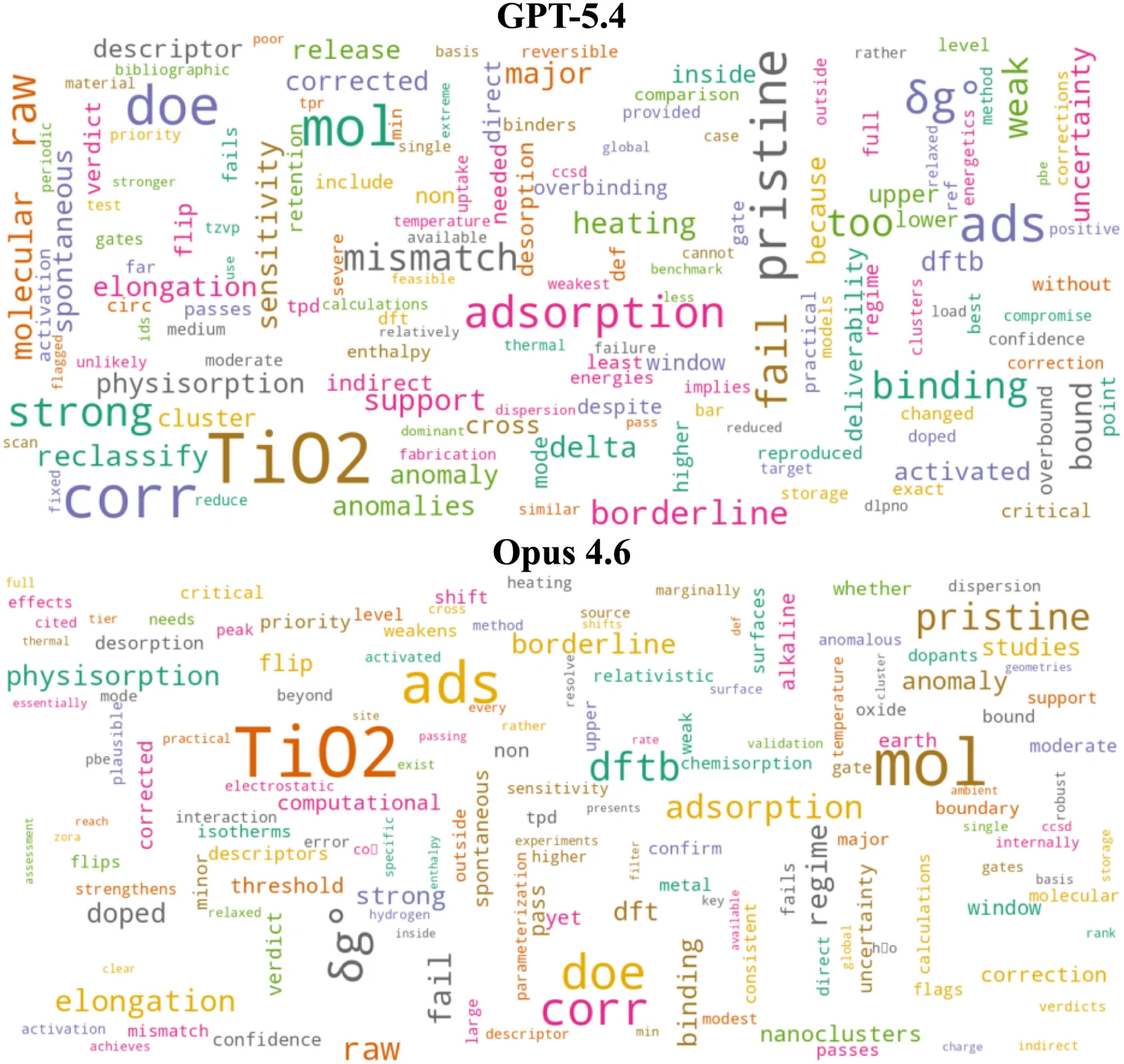
Vocabulary distributions from independent LLM-assisted screening runs. Results are shown for (a) GPT-5.4 and (b) Claude Opus 4.6 (two of the three benchmarked models; agreement across all three is reported in Fig. S3), each based on five independent screening runs for alkaline-earth-modified TiO_2_ nanoparticles. Term size is proportional to within-model frequency. The shared prominence of terms such as adsorption, binding, Δ*G*°, and DFTB-related descriptors indicates substantial consistency in the interpretive vocabulary, whereas differences in term prominence reflect model-specific emphasis.

Detailed multi-model results, including median rank positions, Kendall's *τ* agreement metrics, anomaly detection rates, and DFTB+ flip-verdict statistics, are provided in the SI.

### 3.6 Multi-model consistency and uncertainty analysis

Fig. S3(a) shows that the ranking is highly stable across the three models, with pristine TiO_2_ and Mg–TiO_2_ consistently identified as the most balanced adsorption cases within the present series. Under the entropy-consistent treatment, all three models produce an identical ordering—pristine TiO_2_ (1) and Mg–TiO_2_ (2) at the top, the weak-binding Ca–, Sr–, and Ba–TiO_2_ next (3–5), and the strongly over-binding Ra–TiO_2_ (6) and Be–TiO_2_ (7) at the bottom. The earlier inter-model disagreement over the placement of Ra–TiO_2_ disappears once the broad electronic adsorption-energy window is applied as a hard gate: all three models now penalize its excessive binding consistently and rank it second-lowest, just above Be–TiO_2_. This regime-level consistency indicates that the overall screening outcome is determined primarily by the physics-gated descriptors rather than by model-specific narrative preferences.

The agreement matrix in Fig. S3(b) indicates complete cross-model consistency: all three models produce identical median rankings, giving perfect pairwise agreement (Kendall's *τ* = 1.00 for every model pair). The lower agreement of Opus 4.6 reported previously, which had been driven solely by its higher placement of the strong-binding Ra–TiO_2_, is removed under the entropy-consistent gate definitions. All three models preserve the same physically meaningful regime separation. The robustness-confidence panel in Fig. S3(c) shows that the systems closest to the gate thresholds—pristine TiO_2_ and Mg–TiO_2_ (the most balanced strong-binding tier)—are generally assigned lower confidence than the clearly strong-binding (Be– and Ra–TiO_2_) and weak-binding (Ca–, Sr–, Ba–TiO_2_) systems, consistent with their proximity to the thermodynamic decision boundaries.

The uncertainty-oriented panels in Fig. S3(d)–(f) provide additional context. Across Opus 4.6 and GPT-5.4, nearly every run flags at least one cross-descriptor anomaly for most systems, with the largest anomaly counts for the strong binders Mg–, Ra–, and (in GPT-5.4) Be–TiO_2_, where multiple descriptors jointly indicate excessive binding; Gemini 3.1 Pro is more conservative in its anomaly flagging. The highest flip-verdict rates, by contrast, are associated with pristine TiO_2_ and Mg–TiO_2_, reflecting their proximity to the thermodynamic screening boundary and pinpointing where modest energetic shifts could alter the final classification. Literature support is strongest for pristine TiO_2_ and Mg–TiO_2_, predominantly indirect for Ca–, Sr–, and Ba–TiO_2_, and minimal for Be– and Ra–TiO_2_. Taken together, these panels indicate that the interpretive layer reproduces consistent rankings and localizes uncertainty and evidential support in a way that remains aligned with the underlying thermodynamic picture.

#### 3.6.1 Comparison with literature

Published hydrogen physisorption energies on bulk oxide surfaces are generally much weaker than those computed here. For example, the isosteric heat of adsorption on mesoporous silica is only ∼3–4 kJ mol^−1^,^39^ and quantum liquid-density functional calculations for honeycomb ZnO monolayers give ∼3.2 kJ mol^−1^.^40^ Spillover hydrogen on Au/TiO_2_ has been measured at −7 ± 1 kJ mol^−1^.^41^ In contrast, density-functional theory for FeO surfaces yields adsorption energies in the range −0.45 to −0.5 eV (≈−43 to −50 kJ mol^−1^) for most surfaces, with the (111)-O surface showing only about −0.2 eV (≈−20 kJ mol^−1^).^42^ These values indicate that low-coordination sites can support much stronger binding than bulk surfaces. In our TiO_2_ nanoparticle, the pristine, Mg- and Ra-modified models give DFTB+ adsorption energies of −48 to −66 kJ mol^−1^, Ca/Sr/Ba modifications yield −17 kJ mol^−1^. The Be-modified model displays an exceptionally strong interaction. Among the alkaline-earth surface modifiers considered here, Be has empty 2p orbitals that can hybridize with the *σ* and σ* orbitals of H_2_ to form a Kubas-type dihydrogen complex.^43^ This interaction transfers charge into the H–H antibonding orbital, with H–H bond lengths of ∼0.86 Å reported for such Be(H_2_) complexes (*versus* 0.74 Å in free H_2_) and shortening the Be–H distances to ∼1.5 Å. Such a side-on Be(H_2_) species has a binding energy of roughly 0.3–0.8 eV per H_2_ molecule,^43^ explaining why our Be-modified nanoparticle shows chemisorption rather than weak physisorption. The moderate values around −50 kJ mol^−1^ agree well with those reported for FeO surfaces, supporting the idea that quantum confinement and under-coordinated Ti sites lead to enhanced binding. These comparisons support the physical plausibility of the trends observed across the alkaline-earth series and indicate that they should not be benchmarked only against weak bulk physisorption values. At the same time, we agree that these literature comparisons and the PTBP transferability benchmarks should not be interpreted as a direct adsorption-energy validation for the present H_2_–TiO_2_ nanoparticle complexes. The PTBP parameter set was developed as a broad solid-state baseline parametrization and was benchmarked for elemental solids and multicomponent binary systems, including hydrides and oxides, but not specifically fitted or validated for molecular H_2_ adsorption energies on under-coordinated TiO_2_ nanoparticle sites. Therefore, the adsorption energies reported here are used only as internally consistent DFTB+ screening descriptors. Direct higher-level validation was therefore performed for the pristine and Mg–TiO_2_ representative configurations, which are the systems lying closest to the screening boundaries. As discussed in Section 3.2, PBE-D3(BJ) calculations support attractive H_2_ interaction for both motifs but do not reproduce the DFTB relative energetic ordering. This comparison confirms that the DFTB descriptors are useful for internally consistent screening while also demonstrating that the small pristine/Mg energetic separation is method-sensitive and should not be interpreted as a quantitatively established ranking.

## 4 Conclusion

In this work, we investigated single-H_2_ adsorption on pristine and alkaline-earth-modified anatase TiO_2_ nanoparticles in order to assess how surface modification alters local adsorption thermodynamics under near-ambient conditions. The combined structural, energetic, and finite-temperature screening results show a clear separation of regimes across the series: Be–TiO_2_ and Ra–TiO_2_ lie on the strong-binding side, Ca–, Sr–, and Ba–TiO_2_ remain weak-binding and under-loaded, whereas pristine TiO_2_ and Mg–TiO_2_ occupy the most balanced region of the representative adsorption landscape within the present DFTB-based representative single-site model. The near-invariance of the frontier-orbital and conceptual-DFT descriptors further indicates that the observed differences are governed primarily by local adsorption geometry and adsorption thermodynamics rather than by major global electronic-structure reorganization. Within the DFTB screening model, pristine TiO_2_ and Mg–TiO_2_ emerge as the two most balanced representative cases. Targeted PBE-D3(BJ)/def2-SVP benchmarks confirm attractive H_2_ interaction for both motifs but do not preserve the DFTB pristine/Mg energetic ordering, demonstrating that their relative ranking is method-sensitive. Accordingly, no method-independent energetic preference between pristine and Mg–TiO_2_ is claimed. At the same time, the present framework should be interpreted as a screening-level description of local representative-site binding behavior rather than a direct predictor of storage capacity, and representative systems should be examined in future work using multiple nanoparticle sizes, experimentally motivated faceted and partially crystalline/core–shell morphologies, multi-site adsorption sampling, full-nanoparticle and multi-site higher-level electronic-structure calculations, surface-defect models, and coverage-dependent adsorption conditions.

## Conflicts of interest

There are no conflicts to declare.

## Supplementary Material

RA-OLF-D6RA04252J-s001

## Data Availability

All data generated and analyzed during this study, together with the complete implementation of the GATOR framework, are publicly available at https://github.com/KurbanIntelligenceLab/GATOR-Framework. The repository contains the full source code, accompanying documentation, and representative example workflows, enabling transparency, reproducibility, and reuse of the framework. Supplementary information (SI): thermodynamic derivations and descriptor definitions, supporting structural and energetic data, higher-level DFT benchmark results, detailed screening outputs, and multi-model consistency analyses. See DOI: https://doi.org/10.1039/d6ra04252j.
