# Supplementary material for: Physics-guided thermodynamic screening of H2 adsorption on alkaline-earth-modified anatase TiO2 nanoparticles with LLM-assisted interpretation
Source: RSC Adv. 2026 Oct 7;16(46):60800–13. doi: 10.1039/d6ra04252j (PMC13647219; doi:10.1039/d6ra04252j)
Supplement: Supplementary file 1 [file RA-016-D6RA04252J-s001.pdf]

Supplementary Information (SI) for RSC Advances.
This journal is © The Royal Society of Chemistry 2026

Supplementary Information

for

Physics-Guided Thermodynamic Screening of H_2 Adsorption on Alkaline-Earth-Modified Anatase TiO_2 Nanoparticles with LLM-Assisted Interpretation

Mustafa Kurban[a,b,*], Can Polat[c], Erchin Serpedin[c], Hasan Kurban[d,*]

[a] *Department of Electrical and Computer Engineering, Texas A&M University at Qatar, Doha, Qatar*
[b] *Department of Prosthetics and Orthotics, Ankara University, Ankara, Turkey*
[c] *Department of Electrical and Computer Engineering, Texas A&M University, College Station, TX, USA*
[d] *College of Science and Engineering, Hamad Bin Khalifa University, Doha, Qatar*

S1. Supplementary Structural, Energetic, Electronic, and Validation Data

This section provides the structural, energetic, electronic, higher-level validation, and coverage data underlying the screening descriptors and methodological assessments reported in the main text.

S1.1. Total Energies

Table S1 lists the DFTB+ total electronic energies of the bare nanoparticle, gas-phase H_2, and the adsorbed complex at their relaxed geometries; every adsorption energy quoted in the main text follows from these three numbers through $E_{\text{ads}} = E(\text{NP+H}_2) - E(\text{NP}) - E(\text{H}_2)$, with a common gas-phase reference (−19.4268 eV) shared across all systems by construction.

Table S1: Total electronic energies (eV) of the isolated nanoparticle (NP), gas-phase H_2, and the adsorbed complex (NP+H_2) at the relaxed geometries reported in the main text.

System	$E(\text{NP}+\text{H}_2)$	$E(\text{NP})$	$E(\text{H}_2)$
Pristine TiO_2	−18 951.3928	−18 931.4702	−19.4268
Be–TiO_2	−18 955.9539	−18 934.9621	−19.4268
Mg–TiO_2	−18 953.0122	−18 933.0344	−19.4268
Ca–TiO_2	−18 951.6120	−18 931.9800	−19.4268
Sr–TiO_2	−18 951.7491	−18 932.1190	−19.4268
Ba–TiO_2	−18 951.6054	−18 931.9978	−19.4268
Ra–TiO_2	−18 952.0376	−18 931.9221	−19.4268

All values at the DFTB+ level; the gas-phase H_2 reference is identical across systems by construction.

S1.2. Structural Perturbation Induced by AE Surface Modification

The apparent similarity of the full-particle representations in Figure 2 was quantified by rigidly aligning the 269 common Ti/O framework atoms of each relaxed bare AE-modified nanoparticle to the relaxed pristine TiO_2 reference. The resulting displacement metrics are summarized in Table S2. The small global framework RMSDs (0.052–0.055 Å) show that addition of a single surface-bound AE adatom does not induce wholesale reconstruction of the nanoparticle. The larger local RMSDs (0.102–0.154 Å) and maximum displacements of up to 0.204 Å, however, demonstrate a measurable localized relaxation around the anchoring site.

*Corresponding authors: kurbanm@ankara.edu.tr (M. Kurban), hkurban@hbku.edu.qa (H. Kurban).

Table S2: Quantitative structural perturbation of the TiO_2 framework after AE surface modification. RMSD values were evaluated after optimal rigid-body alignment of the 269 common Ti/O framework atoms to the relaxed pristine TiO_2 nanoparticle. The local RMSD includes framework atoms within 4.0 Å of the anchoring surface O atom.

System	Framework RMSD (Å)	Maximum displacement (Å)	Local RMSD (Å)	Anchoring-O displacement (Å)
Be–TiO_2	0.052	0.158	0.118	0.134
Mg–TiO_2	0.055	0.204	0.154	0.191
Ca–TiO_2	0.052	0.135	0.108	0.124
Sr–TiO_2	0.052	0.151	0.110	0.135
Ba–TiO_2	0.052	0.136	0.102	0.119
Ra–TiO_2	0.052	0.145	0.109	0.145

S1.3. Electronic Structure Descriptors

Table S3 reports the full frontier-orbital and conceptual-DFT descriptor set (E_g, IP, EA, χ, η, ω) for the bare and H_2-adsorbed clusters. Consistent with the main-text discussion, every global descriptor varies only in the second decimal place across the alkaline-earth series and shifts by less than 0.02 eV upon H_2 adsorption, reinforcing that differences in adsorption behavior arise from local geometry and charge redistribution rather than from any global electronic-structure reorganization of the nanoparticle.

Table S3: DFTB+ eigenvalue-based and conceptual-DFT descriptors (eV) for pristine and alkaline-earth-modified TiO_2 nanoparticles, before and after H_2 adsorption.

System	$\varepsilon_{\mathrm{HOMO}}$	$\varepsilon_{\mathrm{LUMO}}$	E_g	IP	EA	χ	η	ω
Bare nanoparticles								
Pristine TiO_2	−8.1160	−4.5332	3.5828	8.116	4.533	6.325	1.791	11.165
Be–TiO_2	−8.1195	−4.5392	3.5803	8.120	4.539	6.329	1.790	11.189
Mg–TiO_2	−8.1185	−4.5278	3.5907	8.118	4.528	6.323	1.795	11.135
Ca–TiO_2	−8.1189	−4.5302	3.5887	8.119	4.530	6.325	1.794	11.146
Sr–TiO_2	−8.1185	−4.5303	3.5882	8.118	4.530	6.324	1.794	11.147
Ba–TiO_2	−8.1181	−4.5299	3.5882	8.118	4.530	6.324	1.794	11.146
Ra–TiO_2	−8.1184	−4.5272	3.5912	8.118	4.527	6.323	1.796	11.132
H_2-adsorbed nanoparticles								
Pristine TiO_2–H_2	−8.1165	−4.5277	3.5888	8.116	4.528	6.322	1.794	11.137
Be–TiO_2–H_2	−8.1193	−4.5296	3.5897	8.119	4.530	6.324	1.795	11.143
Mg–TiO_2–H_2	−8.1185	−4.5305	3.5880	8.118	4.530	6.324	1.794	11.148
Ca–TiO_2–H_2	−8.1184	−4.5302	3.5882	8.118	4.530	6.324	1.794	11.147
Sr–TiO_2–H_2	−8.1184	−4.5296	3.5888	8.118	4.530	6.324	1.794	11.144
Ba–TiO_2–H_2	−8.1183	−4.5297	3.5886	8.118	4.530	6.324	1.794	11.144
Ra–TiO_2–H_2	−8.1190	−4.5301	3.5889	8.119	4.530	6.325	1.794	11.145

$E_g = \varepsilon_{\mathrm{LUMO}} - \varepsilon_{\mathrm{HOMO}}$; $\chi = (\mathrm{IP} + \mathrm{EA})/2$; $\eta = (\mathrm{IP} - \mathrm{EA})/2$; $\omega = \chi^2/(2\eta)$. IP and EA are Koopmans-like quantities obtained from the frontier orbitals.

S1.4. Higher-Level DFT Validation

Two nested local clusters were extracted directly from the DFTB-relaxed adsorption geometries to assess the sensitivity of the representative pristine and Mg–TiO_2 adsorption motifs to the electronic-structure method. PBE-D3(BJ)/def2-SVP interaction energies were evaluated using the Boys–Bernardi counterpoise correction. The full-nanoparticle DFTB adsorption energies are included only as screening-level reference values; because the PBE calculations employ reduced local clusters, the two columns should not be interpreted as a one-to-one full-system energy error.

Table S4: Higher-level PBE-D3(BJ)/def2-SVP validation of representative pristine and Mg–TiO_2 H_2 adsorption motifs. PBE interaction energies are counterpoise-corrected values evaluated at DFTB-derived local geometries.

System	Local model	DFTB full-NP E_{ads} (eV)	PBE-D3(BJ) $E_{\mathrm{int}}^{\mathrm{CP}}$ (eV)
Pristine	Ti_4O_8	-0.496	-0.313
Pristine	Ti_6O_{12}	-0.496	-0.350
Mg–TiO_2	Ti_4O_8	-0.551	-0.173
Mg–TiO_2	Ti_6O_{12}	-0.551	-0.138

The PBE values are local-cluster interaction energies and should not be interpreted as full-nanoparticle adsorption energies. Their purpose is to test the sign, approximate magnitude, cluster-size sensitivity, and relative method dependence of the representative adsorption interactions.

Table S5: Selected points from fixed-framework PBE-D3(BJ)/def2-SVP H_2 displacement scans spanning the local adsorption region. R denotes the distance from the H_2 center to the Ti adsorption center for pristine TiO_2 and to Mg for Mg–TiO_2. Raw and Boys–Bernardi counterpoise-corrected interaction energies are given in kcal mol^{-1}.

System	R (Å)	Raw interaction	CP-corrected interaction
Pristine	1.948	-6.33	-5.21
Pristine	2.148	-8.19	-8.07
Pristine	2.348	-7.98	-9.15
Mg–TiO_2	2.137	-5.10	-4.43
Mg–TiO_2	2.337	-8.22	-7.69
Mg–TiO_2	2.537	-3.74	-5.35

S1.5. Equilibrium Hydrogen Coverage

Figure S1 maps the equilibrium fractional H_2 coverage θ at 298 K and 1, 10, and 30 bar, obtained from the standard-state free energy through the ideal Langmuir relation: under the entropy-consistent $\Delta S_{\text{loss}} = 8R$, the strong binders Be–, Ra–, pristine, and Mg–TiO_2 all saturate ($\theta \to 1$) even below 1 bar, whereas the weak binders Ca–, Sr–, and Ba–TiO_2 stay essentially empty ($\theta \approx 0$) up to 30 bar; no system falls cleanly within the practical near-ambient regime, with pristine and Mg–TiO_2 the most balanced yet still on the strong-binding side.

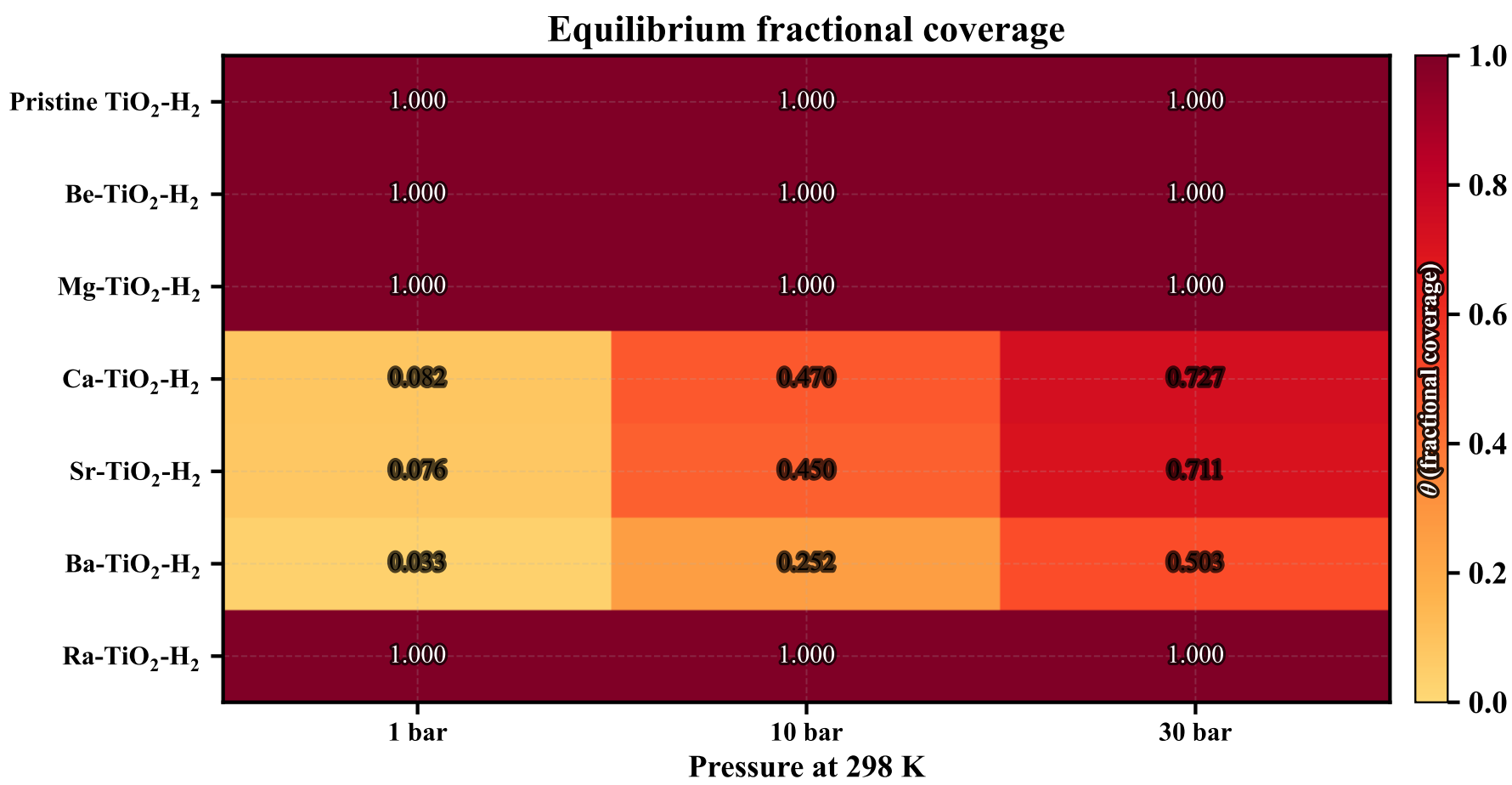

Figure S1: Equilibrium fractional H_2 coverage θ at 298 K for each nanoparticle at 1, 10, and 30 bar. Values were computed from the standard-state free energy and the ideal Langmuir relation, assuming identical, non-interacting adsorption sites. Dark cells ($\theta \to 1$) indicate near-complete site occupancy; light cells ($\theta \approx 0$) indicate negligible uptake. For a heterogeneous nanoparticle surface, these values should be interpreted as first-order screening projections rather than quantitative whole-particle coverages.

S1.6. Adsorption-Induced Descriptor Shifts

Figure S2 quantifies the H_2-induced shifts in band gap (ΔE_g), Mulliken electronegativity ($\Delta\chi$), and absolute hardness ($\Delta\eta$); the uniformly small magnitudes ($\sim 10^{-2}$ eV) across the series confirm that single-H_2 adsorption perturbs the global electronic structure only weakly.

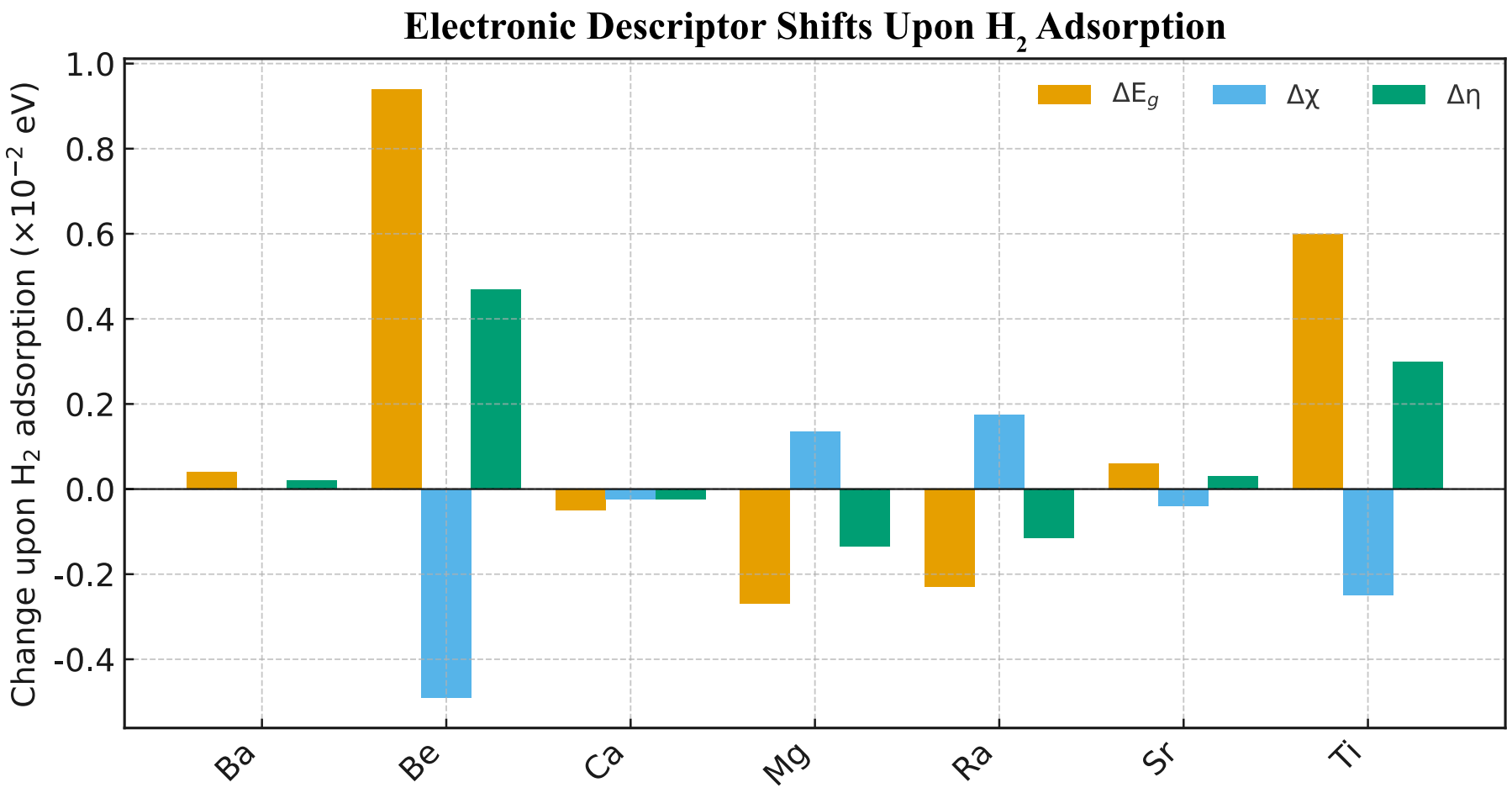

Figure S2: H_2-induced electronic-descriptor shifts relative to the corresponding H_2-free nanoparticle. Grouped bars report the changes in band gap ΔE_g, Mulliken electronegativity $\Delta\chi$, and absolute hardness $\Delta\eta$ upon H_2 adsorption, in units of 10^{-2} eV; positive bars indicate an increase and negative bars a decrease. The uniformly small magnitudes across the series support the interpretation that single-H_2 adsorption perturbs the global electronic structure only weakly.

S2. Extended Details for the LLM-Assisted Screening Framework

S2.1. Stage 1: Deterministic Descriptor Definitions

Electronic and Conceptual-DFT Descriptors from Frontier Orbitals From the DFTB+ frontier orbital eigenvalues $\varepsilon_{\mathrm{HOMO}}$ and $\varepsilon_{\mathrm{LUMO}}$, we compute band-gap and Koopmans-like quantities

$$E_g = \varepsilon_{\mathrm{LUMO}} - \varepsilon_{\mathrm{HOMO}}, \tag{1}$$

$$\mathrm{IP} \approx -\varepsilon_{\mathrm{HOMO}}, \qquad \mathrm{EA} \approx -\varepsilon_{\mathrm{LUMO}}, \tag{2}$$

and the conceptual-DFT descriptors

$$\chi = \frac{\mathrm{IP} + \mathrm{EA}}{2}, \qquad \eta = \frac{\mathrm{IP} - \mathrm{EA}}{2}, \qquad \omega = \frac{\chi^2}{2\eta}, \tag{3}$$

with standard safeguards for near-zero η.

Adsorption Energetics and Heuristic Regime/DOE Classification Electronic adsorption energies are computed from DFTB+ total energies as

$$E_{\mathrm{ads}} = E_{\mathrm{NP+H_2}} - E_{\mathrm{NP}} - E_{\mathrm{H_2}}, \tag{4}$$

with unit conversion $E_{\mathrm{ads}}^{(\mathrm{kJ/mol})} = 96.485\, E_{\mathrm{ads}}^{(\mathrm{eV})}$. An adsorption-strength regime label is assigned via fixed thresholds on $|E_{\mathrm{ads}}^{(\mathrm{kJ/mol})}|$, and a broad raw-electronic screening flag is set based on whether $19.3 \leq |E_{\mathrm{ads}}^{(\mathrm{kJ/mol})}| \leq 57.9$, i.e. $|E_{\mathrm{ads}}| = 0.2$–0.6 eV, corresponding to the broad electronic adsorption-energy window commonly used for reversible H_2 binding. This raw-electronic window is kept distinct from the DOE/HyMARC finite-temperature–corrected enthalpy window (15–25 $\mathrm{kJ\,mol^{-1}}$), which is applied only to ΔH_{corr}.

Geometry-based Adsorption Metrics and Mode Classification From the optimized xyz geometries, we compute the intramolecular H–H bond length $r_{\mathrm{H-H}}$ (Å) and its elongation relative to gas-phase H_2,

$$\Delta r_{\mathrm{H-H}} = r_{\mathrm{H-H}} - r_{\mathrm{H-H}}^{\mathrm{gas}}, \qquad r_{\mathrm{H-H}}^{\mathrm{gas}} = 0.741\ \text{Å}. \tag{5}$$

A discrete adsorption-mode label is assigned using hard thresholds:

$$\mathrm{mode} = \begin{cases} \text{molecular}, & r_{\mathrm{H-H}} < 0.80\ \text{Å} \\ \text{activated}, & 0.80 \leq r_{\mathrm{H-H}} \leq 0.95\ \text{Å} \\ \text{dissociative}, & r_{\mathrm{H-H}} > 0.95\ \text{Å}. \end{cases} \tag{6}$$

For AE-modified systems, additional geometry indicators are computed, including the shortest H–AE distance and nearest O–AE separation.

Finite-temperature Thermodynamic Projections and Operating-point Metrics Two finite-temperature–corrected enthalpy surrogates are formed as

$$\Delta H_{\text{corr}} = E_{\text{ads}}^{(\text{kJ/mol})} + \delta_{\text{corr}}, \qquad \delta_{\text{corr}} \in \{6.0,\ 25.96\}\ \text{kJ/mol}, \tag{7}$$

where δ_{corr} is a zero-point-and-thermal *enthalpy* correction (not an entropy term): the lower value (+6 kJ/mol) is the deciding molecular-physisorption estimate, and the upper value (+25.96 kJ/mol) is an extreme bound of order the full gas-phase H_2 zero-point energy (∼26 kJ/mol), reported only as a sensitivity. The surrogates are mapped to DOE-style labels:

$$\text{DOE} = \begin{cases} \text{Inside (15–25)}, & 15 \le |\Delta H_{\text{corr}}| \le 25 \\ \text{Outside–weak}, & |\Delta H_{\text{corr}}| < 15 \\ \text{Outside–strong}, & |\Delta H_{\text{corr}}| > 25 \\ \text{Endothermic}, & \Delta H_{\text{corr}} > 0. \end{cases} \tag{8}$$

Both corrections are tabulated, but the deciding finite-temperature–corrected enthalpy gate uses the lower-bound +6 kJ mol^{-1} zero-point/thermal correction, which reproduces the committed T_{50} and ΔG° values used throughout the figures and main text; the +25.96 kJ mol^{-1} scheme is an extreme upper bound of order the gas-phase H_2 zero-point energy, reported only as a robustness sensitivity. The δ_{corr} term is an enthalpy correction; the entropy of adsorption enters exactly once, through ΔS_{loss} in the T_{50} and ΔG° expressions below, and is therefore not double-counted. Using an entropy-loss approximation $\Delta S_{\text{loss}} \approx -8R = -0.06651$ kJ/(mol · K) and $R = 0.008314$ kJ/(mol · K), we compute the desorption midpoint temperature

$$T_{50}(p) = \frac{\Delta H_{\text{corr}}}{\Delta S_{\text{loss}} + R \ln p}, \tag{9}$$

for $p = 1$ and 30 bar, and the standard-state free-energy surrogate at 298 K:

$$\Delta G^\circ(298\,\text{K}) = \Delta H_{\text{corr}} - 298\,\Delta S_{\text{loss}}. \tag{10}$$

The same entropy term, $\Delta S_{\text{loss}} \approx -8R$, is applied uniformly to every system. This single, internally consistent desorption-entropy assumption is chosen so that the canonical DOE/HyMARC 15–25 kJ mol^{-1} enthalpy window maps onto $T_{50} \approx$ 226–376 K at 1 bar, which overlaps the practical T_{50} = 250–400 K operating window; the two thermodynamic criteria are thereby internally consistent. It replaces the earlier full gas-phase value ($\Delta S_{\text{loss}} \approx -S^\circ(H_2) \approx -15.7R$), under which the same enthalpy window mapped to $T_{50} \approx$ 115–191 K and was therefore mutually inconsistent with the operating window. Because the enthalpy correction and the entropy penalty act as system-independent shifts, the system ordering and the strong/weak regime separation are unchanged; under the entropy-consistent treatment pristine TiO_2 and Mg–TiO_2 move from the near-deliverable description to the strong-binding (heating-needed) side while remaining the most balanced cases in the series. When finite-T projections are missing for any system, they are deterministically recomputed from $E_{\text{ads}}^{(\text{kJ/mol})}$ using the same definitions above.

S2.2. GATOR Framework: Gate-Augmented Thermodynamic Ordering and Retrieval

The GATOR framework is a physics-constrained, multi-stage screening pipeline designed to evaluate H_2 adsorption on alkaline-earth-modified TiO_2 nanoparticles by combining descriptor-based thermodynamics with retrieval-augmented large language model analysis. The workflow begins with a structured input of DFTB+-derived descriptors for each system, including adsorption energy, H–H bond length, finite-temperature–corrected enthalpy, desorption midpoint temperature, and standard-state free energy. These descriptors are passed through a sequence of six deterministic physics gates that encode established screening criteria: (i) adsorption mode based on $r_{\text{H-H}}$, (ii) binding regime based on $|E_{\text{ads}}|$, (iii) a broad raw-electronic screening window ($|E_{\text{ads}}|$ = 0.2–0.6 eV) on raw adsorption energies, (iv) a DOE/HyMARC finite-temperature–corrected adsorption-enthalpy window using ΔH_{corr} (deciding +6 correction), (v) deliverability via the T_{50} window at 1 bar, and (vi) spontaneity via the sign of ΔG°. Because T_{50} and ΔG° are both linear functions of the single finite-temperature–corrected enthalpy ΔH_{corr} (collinear with the enthalpy gate rather than statistically independent), gates (v) and (vi) act as derived

diagnostics that *flag* rather than *fail*; the disqualifying decisions are carried by the genuinely independent descriptor axes—$r_{\mathrm{H-H}}$ (mode), $|E_{\mathrm{ads}}|$ (binding regime and the broad electronic window, the latter failing when $|E_{\mathrm{ads}}|$ lies outside 0.2–0.6 eV), and $|\Delta H_{\mathrm{corr}}|$ (the enthalpy window). Each gate produces a categorical verdict (pass, flag, fail), and these outcomes are aggregated using a strict rule-based hierarchy in which any fail yields a global FAIL classification, any flag in the absence of fails yields BORDERLINE, and only systems passing all gates are assigned PASS. The resulting gate-by-gate verdicts for all seven systems are reported in Table S6; no system passes every gate, so BORDERLINE—realized only by pristine and Mg–TiO_2—is the highest tier in this series.

Following classification, the pipeline branches according to thermodynamic relevance. Systems labeled as BORDERLINE are routed to a physics-aware retrieval-augmented generation (RAG) stage, where domain-specific literature is retrieved using dense vector embeddings (sentence-transformers) from a curated ChromaDB index, typically with top-k = 5 passages per system. In contrast, FAIL systems bypass retrieval entirely and propagate only their immutable gate verdicts forward, ensuring that clearly non-viable candidates are not artificially promoted by contextual information. The retrieved literature and gate outputs are then assembled into a structured prompt with three components: (i) immutable thermodynamic facts (locked to prevent modification), (ii) supporting literature context, and (iii) task-specific synthesis instructions.

A constrained LLM operates on this structured input using a two-stage execution protocol. In the first call, the model produces a structured analysis (e.g., JSON format) capturing system-wise evaluation, ranking, and justification grounded strictly in the provided descriptors and literature. In the second call, this structured output is converted into a narrative synthesis that includes comparative analysis, constrained ranking, and experimentally relevant insights. Importantly, ranking is explicitly constrained such that BORDERLINE systems must be ordered above FAIL systems, preserving the integrity of the gate-based classification. This design ensures that the LLM acts as an interpreter of physically validated data rather than a generator of unconstrained hypotheses.

The final stage of the GATOR pipeline consists of a post-hoc validation module that enforces consistency and reliability of the generated outputs. This module performs four independent checks: (i) structural validation to confirm the presence of required analysis sections, (ii) ranking validation to ensure compliance with gate-imposed ordering constraints, (iii) numerical validation comparing reported values against source descriptors within a predefined tolerance of 1%, and (iv) citation validation to verify that referenced literature identifiers fall within the retrieved set. Only outputs passing all validation layers are accepted as final screening reports. This integrated design ensures that the overall framework maintains physical fidelity, reproducibility, and transparency, while enabling scalable and uncertainty-aware interpretation of thermodynamic screening results. Figure S3 summarizes the reproducibility of this interpretive layer across the three benchmarked models (Opus 4.6, Gemini 3.1 Pro, GPT-5.4), reporting median per-system rank, pairwise and self-agreement (Kendall's τ), anomaly-detection and verdict-flip rates, and literature-support strength; the models converge on pristine and Mg–TiO_2 as the most balanced cases (the highest-ranked, borderline tier) and reproduce the strong/weak regime separation.

Table S6: Gate-by-gate screening verdicts for all seven systems. Each gate returns `pass`, `flag`, or `fail`, and the overall verdict aggregates them by a strict rule: any *fail* yields FAIL, any *flag* with no fail yields BORDERLINE, and only an all-*pass* system would be labelled PASS. A flag is a caution—a descriptor lying just outside a target window—not a disqualification. The collinear T_{50} (Deliv.) and ΔG° (Spont.) diagnostics flag rather than fail. No system passes every gate, so BORDERLINE (here Pristine and Mg–TiO_2) is the highest realized tier. Pristine and Mg–TiO_2 pass the broad raw-electronic adsorption-energy window and remain spontaneous; their flags arise on the finite-temperature–corrected adsorption-enthalpy window and the deliverability diagnostic (heating-needed, $T_{50} > 400$ K), and for Mg–TiO_2 additionally on the binding regime. The five FAIL systems are each disqualified by an independent hard gate—the enthalpy window (Ca/Sr/Ba, too weak), the binding regime (Be, too strong), or the broad electronic window (Ra and Be, $|E_{\mathrm{ads}}| > 0.6$ eV). Gate definitions and thresholds are given in the Methods and in `screening_config.yaml` (Sec. S2.3); per-system descriptor values appear in the main-text adsorption and finite-temperature tables.

**System**	**Mode**	**Regime**	**DOE_{raw}**	**DOE_{corr}**	**Deliv.**	**Spont.**	**Overall**
Pristine	pass	pass	pass	flag	flag	pass	**BORDERLINE**
Mg–TiO_2	pass	flag	pass	flag	flag	pass	**BORDERLINE**
Ra–TiO_2	pass	flag	fail	flag	flag	pass	FAIL
Be–TiO_2	flag	fail	fail	flag	flag	pass	FAIL
Ca–TiO_2	pass	pass	pass	fail	flag	flag	FAIL
Sr–TiO_2	pass	pass	pass	fail	flag	flag	FAIL
Ba–TiO_2	pass	fail	fail	fail	flag	flag	FAIL

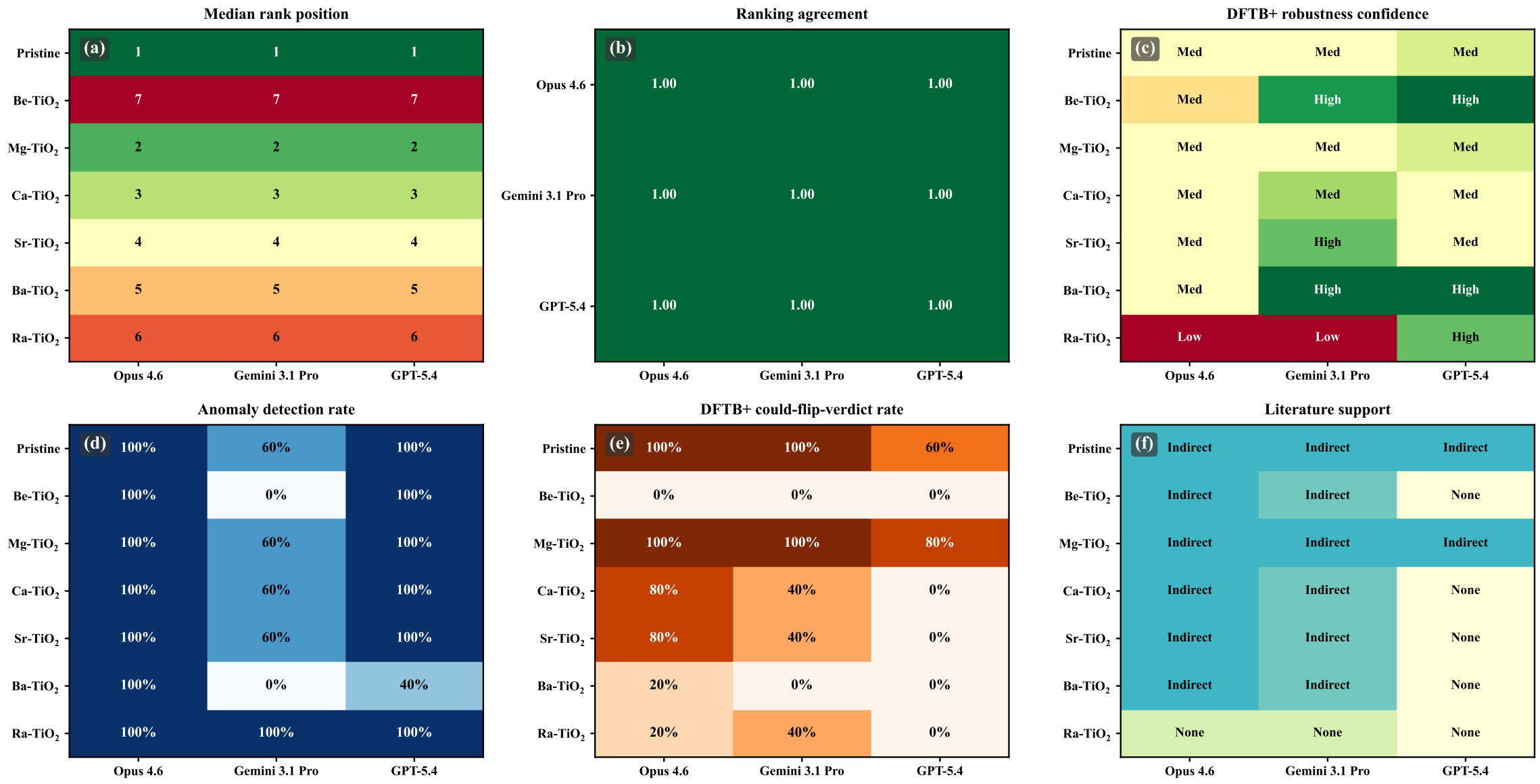

Figure S3: Multi-model structured-analysis benchmark for LLM-assisted H_2-adsorption screening. Three models (Opus 4.6, Gemini 3.1 Pro, GPT-5.4) were each run five times with identical gate verdicts and retrieval context. **(a)** Median rank position per system per model; green indicates top candidates, red lowest-ranked. **(b)** Pairwise and self-agreement measured by Kendall's τ; diagonal entries report within-model consistency, off-diagonal entries inter-model agreement. **(c)** DFTB+ robustness confidence (low/medium/high) reflecting each model's assessment of verdict stability. **(d)** Anomaly-detection rate, i.e., the fraction of runs flagging at least one cross-descriptor anomaly per system. **(e)** DFTB+ could-flip-verdict rate, i.e., the fraction of runs identifying a system as sufficiently close to a gate threshold that method uncertainty could alter the classification. **(f)** Literature-support strength (direct/indirect/none) according to how strongly the retrieved literature supports each system's computed descriptors.

S2.3. LLM Prompt Templates and Screening Configuration

To make the interpretive layer fully reproducible without consulting the source repository, this subsection reproduces the prompt templates passed to the language model and the complete gate-configuration file.

Prompt structure. Every LLM call is assembled deterministically from three sections joined in a fixed order — **(1) immutable facts**, **(2) supporting literature**, and **(3) task instructions** — by the `build_*_prompt` routines in `screening_agent.py`. Section 1 is locked: it holds the deterministic gate verdicts and the key descriptors (the per-system descriptor values are exactly those reported in the main-text adsorption and finite-temperature tables), and the instructions forbid the model from overriding them. The reported results use the two-call structured protocol: Call 1 returns a JSON analysis (Listing S2), and Call 2 converts that JSON into the narrative report (Listing S3); a single-call variant that emits the narrative directly is also available in the code. The overall template skeleton is shown in Listing S1; angle-bracketed fields are filled at runtime from the gate engine, the descriptor table, and the retrieval stage. For typesetting, the few Unicode symbols in the original prompts and configuration are written in ASCII in the listings below (`<=` for $\leq$, `->` for the arrow, `T50` for T_{50}, `Delta_G`/`Delta_H` for $\Delta G/\Delta H$, `Ang` for Å, and `pass/flag/fail` for the gate-verdict marks); the byte-exact files are available in the source repository.

```
# SECTION 1: IMMUTABLE FACTS

Gate verdicts below are deterministic and FINAL. Your analysis must respect them.

| System | Mode | Regime | DOE_raw | DOE_corr | T50 window | Delta_G<0 | Overall |
  <one row per system; verdict cells are "label verdict", verdict in {pass, flag, fail}>

| System | E_ads (kJ/mol) | Delta_H_corr(+6) (kJ/mol) | T50 (K) | Delta_G (kJ/mol) | r_H-H (Ang) | Elong
    % |
  <one row per system>

---

# SECTION 2: SUPPORTING EXPERIMENTAL LITERATURE
  (BORDERLINE systems only; replaced by a "no literature" notice for FAIL systems)

The following experimental results were retrieved from published literature.
Use them to contextualize the computed predictions.
```

```
  <retrieved passages, grouped per system, with citation ids [1]..[N]>

---

# SECTION 3: INSTRUCTIONS   (single-call variant)

Role: computational chemist, H2 adsorption on oxide nanoclusters.
Output: Markdown with ## A, ## B, ## C, ## References. Target <=800 words.
Do not fabricate references or data.

## A. Comparative Analysis      (E_ads / T50 vs literature; DFTB+ uncertainties; coverage gaps)
## B. Constrained Ranking       (BORDERLINE tier above FAIL tier -- mandatory gate constraint)
## C. Experimental Suggestions  (TPD conditions; higher-level calculations)
## References                   (reproduce [1]..[N] from Section 2)
```

Listing S1: Prompt template skeleton (Sections 1–3). Angle-bracketed fields are populated at runtime.

In the two-call protocol used for the reported results, Section 3 is replaced by the two blocks below. Call 1 (Listing S2) requests a machine-readable analysis validated against a fixed JSON schema; Call 2 (Listing S3) turns that analysis into the final narrative.

```
# SECTION 3: STRUCTURED ANALYSIS TASK

Role: computational chemist, H2 adsorption on oxide nanoclusters.
Return ONLY a JSON object matching the schema below. No text outside the JSON.

Tasks:
1. CROSS-DESCRIPTOR ANOMALY DETECTION -- flag systems where descriptors conflict
   (e.g. strong E_ads but weak elongation, or molecular mode with high binding).
2. SENSITIVITY ANALYSIS -- for each borderline system, identify which gate threshold
   shift would flip its verdict.
3. DFTB+ ROBUSTNESS -- assess whether DFTB+ method uncertainty could change the
   screening outcome for each system.
4. LITERATURE GROUNDING -- compare computed values against experimental data
   from Section 2 (if available).
5. RANKING -- order systems from most to least promising for H2 storage,
   respecting the gate constraint that non-fail systems rank above fail systems.

## Output Schema

‘‘‘json
<structured-analysis JSON schema injected here; full schema in output_schemas.py>
‘‘‘
```

Listing S2: Call 1 — structured-analysis instruction block (Section 3).

```
# SECTION 3: INSTRUCTIONS

Role: computational chemist, H2 adsorption on oxide nanoclusters.
Output: Markdown with ## A, ## B, ## C, ## References. Target <=800 words.
Do not fabricate references or data.

## A. Comparative Analysis
1. Discuss EVERY anomaly flag from your structured analysis
2. Compare computed E_ads, T50 against literature values where available
3. Flag DFTB+ method uncertainties that could flip a verdict
4. Note which systems lack experimental support

## B. Constrained Ranking
1. Reproduce the ranking from your structured analysis exactly
2. Justify each position with descriptor values (E_ads, Delta_H_corr, Delta_G, T50)
3. Flag sensitivity: "X would reclassify if threshold Y -> Z"

## C. Experimental Suggestions
1. Prioritize suggestions for systems where dftb_robustness.could_flip_verdict is true
2. TPD conditions for borderline candidates (T, p, heating rate)
3. Higher-level calculations to reduce DFTB+ uncertainty (method, basis)

## References
Reproduce any cited references from Section 1.
```

Listing S3: Call 2 — narrative-synthesis instruction block (Section 3).

Screening configuration. Listing S4 reproduces `configs/screening_config.yaml` in full — the single source of truth for every gate threshold, physics label, and pass/flag/fail mapping, together with the retrieval settings. Changing a threshold is a one-line edit; adding a gate is a single additional block.

```
# GATOR Screening Configuration
# Gate-Augmented Thermodynamic Ordering and Retrieval
#
# All thresholds, labels, and mappings are configurable.
# Physics labels match the vocabulary from labels.csv.
# Screening verdicts (pass/flag/fail) are a separate configurable overlay.

gates:
  - name: adsorption_mode
    description: "H-H bond activation classification"
    column: r_H-H_A
    type: range_multi
    thresholds: [0.80, 0.95]           # Ang -- from adsorption_metrics.py constants
    physics_labels:
      - molecular                       # r_H-H < 0.80 Ang
      - activated                       # 0.80 <= r_H-H <= 0.95 Ang
      - dissociative                    # r_H-H > 0.95 Ang
    screening_map:
      molecular: pass
      activated: flag
      dissociative: fail

  - name: regime
    description: "Adsorption strength regime from |E_ads|"
    column: E_ads_kJ_mol
    type: range_multi
    use_magnitude: true                 # apply abs() before comparison
    thresholds: [18, 50, 100]           # kJ/mol -- from classify_regime() in adsorption_energies.py
    physics_labels:
      - "weak physisorption"            # |E_ads| < 18
      - "moderate physisorption"        # 18 <= |E_ads| < 50
      - "strong physisorption / activated"  # 50 <= |E_ads| < 100
      - "very strong / likely dissociative" # |E_ads| >= 100
    screening_map:
      "weak physisorption": fail
      "moderate physisorption": pass
      "strong physisorption / activated": flag
      "very strong / likely dissociative": fail

  - name: doe_raw
    description: "Broad raw-electronic E_ads screening window (|E_ads| = 0.2--0.6 eV); distinct from the
     DOE/HyMARC enthalpy window"
    column: E_ads_kJ_mol
    type: doe_window
    use_magnitude: true
    doe_bounds: [19.3, 57.9]            # kJ/mol -- 0.2--0.6 eV x 96.485 (ELECTRONIC_WINDOW_MIN/MAX)
    physics_labels:
      inside: "Inside (19.3--57.9)"
      outside: "Outside"
    screening_map:
      "Inside (19.3--57.9)": pass
      "Outside": fail    # R2: |E_ads| outside 0.2--0.6 eV disqualifies

  - name: doe_corrected
    description: "DOE/HyMARC finite-temperature-corrected adsorption-enthalpy window on Delta_H_corr (+6
     kJ/mol; deciding correction)"
    column: Delta_H_plus6_kJ_mol
    type: entropy_corrected_doe
    doe_bounds: [15, 25]                # kJ/mol magnitude
    # Logic: value > 0 -> Endothermic; |value| < 15 -> Outside--weak;
    #        |value| in [15,25] -> Inside; |value| > 25 -> Outside--strong
    physics_labels:
      inside: "Inside (15--25)"
      weak: "Outside--weak"
      strong: "Outside--strong"
      endothermic: "Endothermic"
    screening_map:
      "Inside (15--25)": pass
      "Outside--weak": fail
```

```yaml
      "Outside--strong": flag
      "Endothermic": fail

  - name: deliverability
    description: "Desorption midpoint temperature diagnostic (T50 collinear with Delta_H_corr; R2: flags
     , does not fail)"
    column: T50_at_1bar_K
    type: range_multi
    thresholds: [250, 400]              # K -- near-ambient desorption-midpoint window
    physics_labels:
      - "Non-deliverable--under-load"    # T50 < 250 K
      - "Deliverable @ ~298 K"          # 250 <= T50 <= 400 K
      - "Non-deliverable--heating-needed" # T50 > 400 K
    screening_map:
      "Non-deliverable--under-load": flag
      "Deliverable @ ~298 K": pass
      "Non-deliverable--heating-needed": flag

  - name: spontaneity
    description: "Standard free-energy sign diagnostic at 298 K (Delta_G collinear with Delta_H_corr; R2
     : flags, does not fail)"
    column: Delta_G_std_kJ_mol
    type: threshold
    operator: "<"
    value: 0
    physics_labels:
      "true": spontaneous
      "false": non-spontaneous
    screening_map:
      spontaneous: pass
      non-spontaneous: flag

# Aggregation rule for combining gate verdicts into an overall classification
aggregation:
  pass: "all gates pass"
  borderline: "any gate flags, none fail"
  fail: "any gate fails"

# RAG settings
rag:
  top_k: 5
  embedding_model: "all-MiniLM-L6-v2"
  literature_dir: "literature"
```

Listing S4: `configs/screening_config.yaml` (complete).

Run provenance. The reported LLM results were produced with the OpenRouter provider using three models (`anthropic/claude-opus-4.6`, `google/gemini-3.1-pro-preview`, and `openai/gpt-5.4`), decoding temperature 0.2, with five independent runs per model over the seven systems. All fifteen runs passed all four post-hoc validation layers (structural completeness, gate-respecting ranking, numerical agreement within 1% of the source descriptors, and citation-identifier checks) described above.
